# Cuproptosis-triggering nanomedicine boosts antitumor immunotherapy

**DOI:** 10.7150/thno.123080

**Published:** 2026-01-01

**Authors:** Maoshan Wang, Chunyu Shi, Zhenbo Shu, Zhongmin Li

**Affiliations:** Department of Gastrointestinal and Colorectal Surgery, China-Japan Union Hospital of Jilin University, Changchun 130033, P. R. China.

**Keywords:** cuproptosis, nanomedicine, antitumor immunotherapy, combination therapy

## Abstract

Cuproptosis, a copper-dependent programmed cell death triggered by mitochondrial copper accumulation and subsequent proteotoxic stress, has emerged as a promising strategy to enhance antitumor immunity. However, conventional cuproptosis inducers face critical limitations such as short blood circulation half-lives, dose-dependent systemic toxicity, and inadequate tumor targeting‌.‌ To address these challenges, advanced nanoplatforms have been developed to enable precise tumor-targeted cuproptosis induction and immune activation. This review summarizes the immune-activating mechanisms of cuproptosis, including its roles in promoting immune cell maturation and infiltration, remodeling the immunosuppressive tumor microenvironment, modulating immune checkpoint molecule expression, and activating the cyclic GMP-AMP synthase-stimulator of interferon genes (cGAS-STING) pathway. We highlight cutting-edge advancements in nanomaterial-based strategies for triggering cuproptosis, which enhance antitumor immunity whether used as a single treatment or in combination with other antitumor modalities. The current challenges in translating cuproptosis-based therapies into clinical applications are proposed to promote the development of cuproptosis-triggering nanomedicines as next-generation immunotherapy strategy.

## 1. Introduction

Copper, an essential trace element, plays a critical role in various physiological processes. It functions as both a cofactor for enzymes involved in energy metabolism and neurotransmitter synthesis and as a metal allosteric modulator that regulates intracellular signal transduction 1. The cellular uptake of copper ions is primarily mediated by the high-affinity copper transporter 1 (CTR1), which is encoded by the SLC31A1 gene 2. This proton-coupled transport mechanism ensures selective and efficient copper acquisition, with optimal activity observed under slightly acidic conditions. Following internalization, copper is rapidly bound by specific chaperones to mitigate potential oxidative damage. Concurrently, intricate regulatory pathways are activated to maintain copper homeostasis, achieving a critical balance between preventing toxicity and ensuring adequate availability for essential cellular functions. When intracellular copper levels exceed the threshold maintained by cellular homeostatic mechanisms, it becomes cytotoxic and may lead to cell death 3. Although copper overload was known to induce cell death, the underlying mechanism remains unclear. In 2022, Tsvetkov et al. identified "cuproptosis", a novel form of mitochondrial respiration-dependent cell death driven by copper overload 4. This process involves excessive intracellular copper ions binding to lipoylated proteins in the mitochondrial tricarboxylic acid (TCA) cycle, leading to protein aggregation and depletion of iron-sulfur (Fe-S) clusters, which in turn induces proteotoxic stress and ultimately leads to cell death 5. Reactive oxygen species (ROS)-dependent oxidative stress emerges as a critical potentiator in cuproptosis. Disruption of copper homeostasis triggers mitochondrial ROS overproduction, which exacerbates the destabilization of Fe-S clusters and disrupts protein folding, thereby intensifying proteotoxic stress 6. This provides a compelling rationale for integrating cuproptosis inducers with ROS-modulating therapeutic strategies 7. Importantly, while ROS plays a permissive role in sensitizing cells to copper-induced toxicity, it is not an indispensable driver of this process, as evidenced by the inability of classical antioxidants to rescue cells from elesclomol (ES)-copper-induced death 8.

Indeed, tumors exhibit a heightened dependence on copper compared to normal tissues, as evidenced by elevated serum copper levels in cancer patients 9. Increasing evidence implicates cuproptosis-related genes as clinically relevant biomarkers. Overexpression of ferredoxin 1 (FDX1) is associated with improved survival in clear-cell renal carcinoma. Similarly, elevated lipoic acid synthase (LIAS) expression predicts improved clinical outcomes in breast cancer, and upregulated expression of dihydrolipoamide S-acetyltransferase (DLAT) predicts poor prognosis in hepatocellular carcinoma 10, 11. These findings underscore the dual utility of cuproptosis-related molecules as both prognostic biomarkers and therapeutic targets. Yang et al. established a xenograft colorectal cancer model in nude mice to evaluate the antitumor effects of 4-octyl itaconate (4-OI) and ES 12. ES functions as a potent copper ionophore, facilitating the transport of extracellular copper ions across lipid bilayers into cellular compartments while bypassing homeostatic regulatory mechanisms. This selective delivery strategy elevates intratumoral copper levels, thereby inducing cuproptosis in cancer cells 13. 4-OI serves as a cuproptosis sensitizer through the inhibition of aerobic glycolysis. Significant inhibition of tumor growth was observed in the ES group compared to the control group. Furthermore, the combination therapy group (ES + 4-OI) demonstrated even greater suppression of tumor volume than the ES-treated group. Immunohistochemical analysis revealed that tumors from the ES + 4-OI group exhibited reduced proliferative activity, as evidenced by a lower Ki-67 index. These findings demonstrate that ES effectively inhibits tumor cell viability through cuproptosis induction.

Cuproptosis-based therapies offer several unique advantages in oncology. Firstly, they target tumors with specific metabolic vulnerabilities, such as reliance on oxidative phosphorylation or disrupted copper homeostasis, which are common in aggressive cancers like triple-negative breast cancer (TNBC) 14. By targeting cells with high copper transporter expression or defective copper efflux pathways, these therapies selectively kill tumor cells while sparing healthy tissues, showing promising therapeutic efficacy in pancreatic cancer and glioblastoma models 15, 16. Secondly, they bypass resistance to apoptosis-based treatments by triggering a distinct death mechanism involving mitochondrial protein aggregation and Fe-S cluster disruption. FDX1-mediated cuproptosis acts independently of B-cell lymphoma 2 proteins or caspase activation, effectively targeting apoptosis-resistant tumors 17. Finally, the combination of cuproptosis with conventional therapies, including chemotherapy and molecular targeted therapy, has shown synergistic antitumor activity in preclinical models. This multimodal approach not only improves treatment efficacy but also addresses the challenge of drug resistance, a major limitation of current cancer therapies 18. Beyond direct cytotoxicity, cuproptosis also exerts profound immunomodulatory effects. By inducing immunogenic cell death (ICD), it triggers the release of damage-associated molecular patterns (DAMPs), including mitochondrial DNA (mtDNA), calreticulin (CRT), high-mobility group box 1 (HMGB1), and adenosine triphosphate (ATP) 19. These signals promote dendritic cell (DC) maturation and antigen presentation while recruiting and activating cytotoxic T lymphocytes (CTLs) and natural killer (NK) cells, effectively converting immunologically "cold" tumors into "hot" tumors. Cuproptosis also reprograms the immunosuppressive tumor microenvironment (TME) by polarizing tumor-associated macrophages (TAMs) toward a pro-inflammatory M1 phenotype and inhibiting the activity of regulatory T cells (Tregs) 20. The released mtDNA, in particular, activates the cyclic GMP-AMP synthase-stimulator of interferon genes (cGAS-STING) pathway, inducing type I interferon production that amplifies antigen presentation, enhances T cell priming, and establishes long-term immune memory 21. Notably, copper-induced oxidative stress upregulates programmed death-ligand 1 (PD-L1) expression, providing a mechanistic rationale for combining cuproptosis inducers with immune checkpoint inhibitors (ICIs) 22.

Despite its numerous advantages and promising preclinical results, the clinical translation of cuproptosis-based immunotherapeutic strategies faces several challenges. Current cuproptosis inducers, including copper ionophores (such as ES and disulfiram (DSF)) and small-molecule copper modulators, demonstrate poor tumor-targeting capabilities, rapid systemic clearance, and inefficient penetration through dense tumor stroma. These limitations result in subtherapeutic copper concentrations at tumor sites while increasing the risk of off-target toxicity in copper-sensitive normal tissues 23. Furthermore, tumor heterogeneity, characterized by differences in cell morphology, gene expression, and metabolic state, complicates therapeutic efficacy. Monotherapies relying solely on cuproptosis induction often encounter adaptive resistance due to mechanisms such as upregulation of copper efflux pumps, activation of alternative cell death pathways, and metabolic reprogramming in tumor cells 24. Although combination strategies with ICIs or other antitumor modalities have demonstrated encouraging synergy in preclinical models, they often exacerbate systemic toxicity, highlighting the necessity for careful optimization of dosing regimens and therapeutic sequencing.

Nanotechnology presents transformative opportunities to overcome the critical challenges in cuproptosis-enhanced immunotherapy. Engineered nanoparticles enable tumor-specific copper delivery through both passive (enhanced permeability and retention (EPR) effect) and active targeting, resulting in superior intratumoral accumulation compared to small-molecule carriers. Stimuli-responsive nanoplatforms facilitate controlled release of copper ions in response to TME cues, such as acidic pH, elevated ROS and glutathione (GSH), or enzymatic activity, thereby confining cuproptosis to tumor tissues 25. At the cellular level, nanocarriers significantly enhance copper uptake through multiple mechanisms. These include receptor-mediated endocytosis, which facilitates cellular internalization of copper-loaded nanoparticles; biomimetic membrane coatings that mimic biological surfaces to promote cell recognition and uptake; and the employment of copper ionophores such as ES, which increase intracellular copper levels by transporting copper ions across membranes. Furthermore, mitochondria-targeting ligands like triphenylphosphonium (TPP) effectively direct copper ions to TCA cycle enzymes within the mitochondrial matrix. This targeted delivery disrupts mitochondrial homeostasis, thereby initiating cuproptosis. For instance, Xin et al. developed platelet membrane-camouflaged polydopamine (PDA)-coated copper nanoparticles (PDA@Cu), which demonstrated enhanced tumor accumulation capabilities 26. Moreover, surface-adaptive designs, including charge modulation and size-switching, improve the tumor penetration of nanomaterials 27. Nanocarriers also enable the co-delivery of cuproptosis inducers and immunomodulators, thereby synchronizing ICD induction with immunosuppression reversal 28. Multifunctional nanosystems combine cuproptosis with established modalities, such as chemotherapy, radiotherapy (RT), and photodynamic therapy (PDT), to amplify therapeutic outcomes at lower drug doses 29. Importantly, the incorporation of imaging components enables real-time monitoring of copper distribution and therapeutic response, facilitating personalized treatment optimization 30.

Despite the promising preclinical advancements, translating cuproptosis-induced nanomedicines into clinical applications faces significant challenges. Key hurdles include concerns over nanoparticle biocompatibility and potential long-term systemic toxicity, particularly from prolonged exposure to engineered materials 31. The complex *in vivo* environment poses additional obstacles, such as inconsistent circulation stability, immune system recognition, and variable tumor penetration efficiency 32. Challenges also extend to accurate biomarkers for patient stratification, real-time monitoring of copper biodistribution, and robust assessment of treatment efficacy in clinical settings. The scalability and reproducibility of nanoparticle synthesis must be standardized to meet stringent regulatory requirements, thereby addressing potential off-target effects and ensuring safe dosage regimens 33. Finally, the integration of cuproptosis-based therapies with existing cancer treatments requires rigorous evaluation to maximize synergistic benefits while minimizing adverse interactions. Addressing these challenges will be critical to advancing cuproptosis-based nanotherapies toward clinical translation and ultimately improving patient outcomes.

**Scheme 1:**
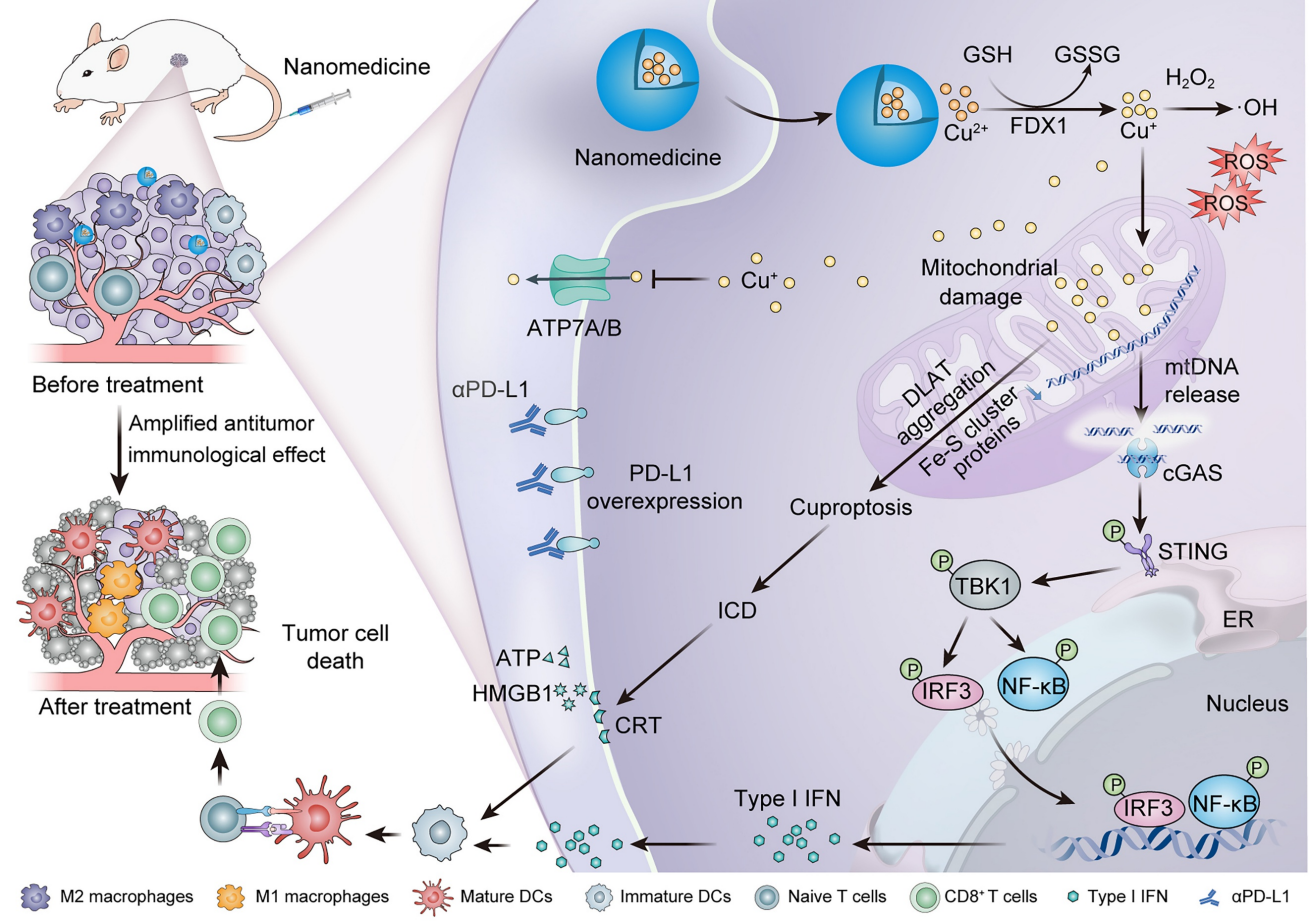
Scheme depicting nanomaterial-mediated enhancement of cuproptosis for tumor immunotherapy.

Herein, we present a comprehensive mechanistic investigation of nanomaterial-mediated cuproptosis as an emerging strategy to potentiate cancer immunotherapy (Scheme 1). This review not only provides an in-depth analysis of innovative nanotechnological approaches for precise copper delivery systems and synergistic immunomodulation strategies but also delineates the fundamental mechanisms underlying this process. Additionally, we highlight key translational challenges, including mechanistic complexity, safety concerns, and scalability issues in large-scale manufacturing. By achieving a seamless integration of biological insight and nanotechnological advancement, we establish a robust framework for developing mechanism-guided, clinically translatable cuproptosis-based cancer immunotherapies with groundbreaking potential.

## 2. Immunomodulatory mechanisms of cuproptosis in antitumor immunity

### 2.1. Promoting immune cell maturation and infiltration

Cuproptosis has emerged as a novel ICD modality capable of robustly activating antitumor immunity through enhanced DC maturation and immune cell infiltration. As a metal-dependent form of regulated cell death, cuproptosis triggers the release of DAMPs, including HMGB1, CRT, and ATP, which serve as critical danger signals to initiate innate and adaptive immune responses 34, 35. These DAMPs function as potent immunomodulators by binding to pattern recognition receptors on antigen-presenting cells, particularly DCs, thereby promoting their phenotypic and functional maturation. Upon DAMP recognition, DCs undergo significant upregulation of co-stimulatory molecules (CD80, CD86) and major histocompatibility complex (MHC) class II expression, enhancing their capacity for tumor antigen presentation 36. Beyond DC activation, cuproptosis activates the immune system by facilitating effector immune cell infiltration 37. The cytokine milieu generated by mature DCs (e.g., CXCL9/CXCL10) promotes the chemotaxis of CTLs and NK cells into tumor beds while suppressing Treg activity 38, 39.

The immunostimulatory effects of cuproptosis-induced ICD have been validated in multiple studies. Li et al. demonstrated that copper ionophore treatment in colorectal cancer cells activates cuproptosis, as evidenced by the significant upregulation of cuproptosis-related markers 35. Western blot and enzyme-linked immunosorbent assay (ELISA) revealed an obvious increase in both the expression and secretion of HMGB1, a hallmark DAMP, in copper ionophore-treated cells compared to the control group. Immunofluorescence staining further confirmed the translocation of CRT to the plasma membrane, a defining feature of ICD. Flow cytometry demonstrated that copper ionophore-treated colorectal cancer cells effectively stimulated DC maturation, as reflected by significantly elevated surface expression of CD80, CD86, and MHC class II molecules. *In vivo* experiments showed a marked enhancement of T-cell infiltration in the tumor following copper ionophore treatment, underscoring the immunostimulatory effects of cuproptosis. Wu et al. successfully synthesized the nanocomposite CuEC@CM-cRGD *via* a one-pot method, which demonstrated potent cuproptosis-inducing effects 40. In a murine tumor model, flow cytometry analysis revealed a significant elevation in the proportions of CD8^+^ T cells in tumor tissues following CuEC@CM-cRGD treatment. Specifically, these immune cells exhibited a 4.7-fold increase compared with the PBS group. Furthermore, the proportion of CD80^+^CD86^+^ DCs in the tumors reached 49.5%, representing a significant increase relative to the PBS control (20.8%). These findings collectively underscore the capacity of CuEC@CM-cRGD to effectively activate immune cells and promote their infiltration in the tumor.

### 2.2. Reshaping the immunosuppressive TME

The efficacy of immunotherapy is often constrained by the immunosuppressive TME. This inhibitory niche, characterized by the infiltration and activity of TAMs, myeloid-derived suppressor cells (MDSCs), and Tregs, collectively hinders antitumor immune responses and promotes tumor immune evasion. Notably, cuproptosis not only enhances immune cell infiltration and activation but also modulates the function of these immunosuppressive populations, thereby potentiating antitumor immunity and offering a promising strategy to overcome TME-mediated immunosuppression 41.

TAMs exhibit remarkable plasticity, existing along a spectrum from the pro-inflammatory M1 phenotype to the anti-inflammatory M2 phenotype. While M1 macrophages secrete pro-inflammatory cytokines (e.g., tumor necrosis factor-α (TNF-α), interleukin-12) and enhance antigen presentation to activate antitumor immunity, M2 macrophages promote tumor progression through the secretion of immunosuppressive factors (e.g., interleukin-10, transforming growth factor-β) and angiogenesis-promoting molecules 42. The dynamic equilibrium between these functionally distinct macrophage populations critically determines the immunological tone of the TME and significantly influences the response to immunotherapy 43. Recent evidence indicates that cuproptosis has the potential to reprogram this immunosuppressive landscape 44. The excessive accumulation of intracellular copper ions during cuproptosis triggers a remarkable phenotypic shift in TAMs, converting immunosuppressive M2 macrophages into immunostimulatory M1 phenotypes 45, 46. This transition is mediated through copper-induced metabolic rewiring and the release of DAMPs, which activate innate immune signaling pathways.

Chang et al. developed an innovative strategy for efficiently encapsulating copper ions in PDA nanostructures (PDA-DTC/Cu), enabling precise delivery of copper ions into cells 47. This design ensures continuous intracellular copper accumulation, ultimately triggering cuproptosis. The immunomodulatory effects of PDA-DTC/Cu nanoparticles were evaluated in a 4T1 tumor-bearing model. Mice were administered intravenous injections of phosphate-buffered saline (PBS), PDA-diethyldithiocarbamate (PDA-DTC), free Cu^2+^, diethyldithiocarbamate-Cu (DTC-Cu), or PDA-DTC/Cu. Flow cytometry analysis revealed that PDA-DTC/Cu nanoparticles significantly upregulated CD80 expression (a marker of M1 macrophages) and downregulated CD206 expression (a marker of M2 macrophages). Specifically, CD206 expression decreased to 5.72% in the PDA-DTC/Cu-treated group, representing an approximately 0.8-fold reduction compared to the 7.1% observed in PBS-treated controls. These results demonstrate that PDA-DTC/Cu nanoparticles effectively repolarize M2 macrophages toward the pro-inflammatory M1 phenotype. Furthermore, PDA-DTC/Cu treatment significantly reduced the proportion of MDSCs from 46.85% in PBS-treated mice to 18.23%, highlighting its robust immunoregulatory effects. These findings collectively establish that PDA-DTC/Cu nanoparticles not only induce cuproptosis but also modulate TAMs and suppress immune suppressive cells, underscoring their potential as a promising therapeutic strategy for cancer immunotherapy. In another study, Ye et al. engineered dynamic nanoparticles (Cu-VT NPs) and demonstrated their profound antitumor effects by inducing cuproptosis 48. Immunofluorescence staining of tumor tissues from nude mouse models treated with Cu-VT NPs revealed a pronounced upregulation of M1 macrophage markers, including CD86 and inducible nitric oxide synthase, alongside a concomitant downregulation of M2 markers such as CD206 and arginase-1. To further validate these findings, tumor tissues were processed into single-cell suspensions, immunolabeled with fluorescently tagged CD206 and CD86 antibodies, and analyzed *via* flow cytometry. This comprehensive analysis confirmed a significant shift in the polarization of TAMs toward the M1 phenotype in the Cu-VT NPs-treated group.

Tregs are pivotal components of the immune system, playing a key role in maintaining immunological homeostasis through suppression of excessive or inappropriate immune responses. However, in the TME, Tregs predominantly exhibit immunosuppressive functions by inhibiting effector T cell activity, thereby impairing antitumor immunity and facilitating tumor progression and metastasis 49. Among the mechanisms underlying Treg-mediated immunosuppression, cytotoxic T-lymphocyte-associated protein 4 (CTLA-4) emerges as a critical regulator. Recent studies demonstrate that cuproptosis significantly reduces Treg proportions and downregulates CTLA-4 expression, thereby improving the tumor immune microenvironment 50, 51. For instance, Xie et al. developed a new polyethylene glycol (PEG)-coated nanomaterial, termed Cu_2_O-MnO@PEG (CMP), that directly induces cuproptosis in tumor cells 52. Through single-cell RNA sequencing and flow cytometry, a substantial reduction in Treg proportions was observed in the tumor tissues of CMP-treated mice compared to the control group. Notably, this was accompanied by a marked decrease in both the transcriptional levels of CTLA-4, the hallmark Treg marker gene, and CTLA-4 protein expression. Furthermore, uniform manifold approximation and projection analysis provided complementary evidence, demonstrating that CMP treatment notably diminished the proportion of Tregs in the T cell-infiltrating population in a subcutaneous melanoma model. These results underscore the importance of targeting cuproptosis as a promising approach for cancer immunotherapy by modulating Treg dynamics in the tumor immune landscape.

MDSCs are a heterogeneous population of immature myeloid progenitors originating from bone marrow hematopoietic stem cells 53. Under physiological conditions, these precursor cells differentiate into mature granulocytes, monocytes, macrophages, and DCs, which collectively contribute to immune defense and surveillance. However, in the TME, MDSCs undergo functional alterations and acquire potent immunosuppressive properties, impairing T cell, NK cell, and other immune effector functions 54. This immunosuppression fosters an environment conducive to tumor growth, proliferation, and metastasis. Cuproptosis has been implicated in enhancing antitumor immunity by reducing MDSC infiltration and alleviating their immunosuppressive effects. Guo et al. developed a ROS-responsive nanoparticle, NP@ESCu, to induce robust cuproptosis of tumor cells 55. *In vivo* experiments revealed that treatment with NP@ESCu significantly reduced MDSC infiltration compared to the control group. The proportion of MDSCs in tumors was markedly lower in NP@ESCu-treated mice (8.4%) versus PBS-treated controls (21.4%). This substantial reduction underscores the capacity of cuproptosis to modulate MDSC dynamics, thereby mitigating their immunosuppressive influence.

The interaction between cuproptosis and other regulated cell death pathways plays a pivotal role in shaping the tumor immune microenvironment 56. Apoptosis, traditionally viewed as non-immunogenic, may acquire immunogenic properties under specific stress conditions 57. This shift enables apoptosis to synergize with cuproptosis, amplifying immune signaling and tumor clearance. Moreover, ferroptosis, a form of iron-dependent cell death, amplifies immune responses in a manner similar to cuproptosis by inducing oxidative stress and ROS release 58. Similarly, pyroptosis shares mechanistic parallels with cuproptosis through its release of pro-inflammatory cytokines and DAMPs. These pathways collectively orchestrate an immunogenic cascade that facilitates immune cell recruitment and activation 59. Engineered nanomaterials offer significant potential for coordinating multiple ICD pathways to enhance cancer immunotherapy. These nanoparticles target distinct molecular vulnerabilities in tumor cells, amplifying oxidative stress and DAMP release while sensitizing cells to apoptosis, thereby maximizing immunogenic potential 24. For instance, ROS-generating nanoagents can trigger both ferroptosis and cuproptosis while sensitizing cells to apoptosis, thereby maximizing immunogenic potential. By controlling DAMP release kinetics and redox states, nanoparticles can repolarize immune suppressive TAMs toward antitumor phenotypes while enhancing T-cell infiltration and activity. This integrated approach underscores the therapeutic potential of regulated cell death pathway crosstalk and highlights the importance of nanomaterial engineering in achieving comprehensive tumor control and overcoming TME-mediated immunosuppression.

Nanomaterial-mediated cuproptosis represents a significant advancement in cancer immunotherapy by targeting the immunosuppressive TME. However, monotherapy with cuproptosis inducers is often insufficient to achieve robust immune regulation 60. Firstly, while cuproptosis inducers have shown efficacy in preclinical models, their therapeutic potential is significantly hampered by the highly adaptive and heterogenous nature of the TME. The intricate crosstalk between various immune cell subsets within the TME often leads to compensatory mechanisms that mitigate the effects of single-agent therapies. Secondly, current cuproptosis-based strategies primarily focus on inducing ICD without adequately addressing the underlying mechanisms driving TME reprogramming. For instance, the role of key immune checkpoints and metabolic regulators in modulating cuproptosis efficiency remains poorly understood. Moreover, the immunosuppressive TME exhibits remarkable plasticity, allowing it to adapt to therapeutic interventions through mechanisms such as upregulation of alternative immune evasion pathways. This dynamic interplay underscores the need for more comprehensive combination strategies that simultaneously target multiple nodes of TME regulation.

Although nanomaterial-mediated cuproptosis offers a novel approach to modulate the immunosuppressive TME, its translation into clinical practice requires addressing several key limitations. These include improving targeting specificity, elucidating underlying mechanisms, and developing rational combination therapies to overcome adaptive resistance. Future studies should focus on integrating multi-omics approaches, advanced nanotechnology, and mechanistic investigations to achieve robust and durable immune remodeling in the clinic.

### 2.3. Regulating immune checkpoint molecule expression

Emerging evidence reveals that cuproptosis dynamically modulates PD-L1 expression through copper-dependent mechanisms, creating unique opportunities for combination immunotherapy 51. The increase in intracellular copper ion concentration may increase PD-L1 expression by up-regulating signal transducer and activator of transcription 3-dependent transcription or inhibiting ubiquitin-proteasome system-mediated PD-L1 degradation, thereby enhancing tumor susceptibility to therapy targeting the programmed cell death 1 (PD-1)/PD-L1 axis 61. Notably, copper ionophores enhance tumor immunogenicity while sustaining PD-L1 expression, thereby augmenting checkpoint blockade efficacy. Nanotechnology-enabled copper delivery systems further optimize this balance by achieving spatiotemporal control of PD-L1 modulation while minimizing systemic toxicity 62. Such approaches hold particular promise in overcoming resistance to PD-1/PD-L1 checkpoint inhibition, especially in immunologically "cold" tumors that traditionally exhibit limited responsiveness to immunotherapy 63.

Liu et al. observed a significant upregulation of PD-L1 expression in the DSF/Cu^2+^-treated group compared to the control group, providing a reference for the combination of cuproptosis and PD-L1 blocking therapy 64. Huang et al. engineered a spherical nucleic acid nanoparticle platform based on an enzyme-catalyzed core (CAT-ecSNA-Cu), specifically designed to effectively induce cuproptosis through targeted copper ion delivery 65. In CT26 tumor-bearing mice, immunofluorescence assays revealed that PD-L1 fluorescence intensity in tumor tissues treated with CAT-ecSNA-Cu was significantly elevated compared to the control group without cuproptosis induction. Further experiments demonstrated that the combination of CAT-ecSNA-Cu and αPD-L1 antibody treatment elicited a robust synergistic immune activation response. Notably, the proportion of mature DCs reached 63.7% in inguinal lymph nodes and 30.3% in the spleen, both significantly higher than those observed in other groups. Moreover, the percentage of CD69^+^CD8⁺ T cells in distant tumor tissues increased to 70.6%, representing a marked enhancement compared to the PBS group (26.4%). These findings collectively indicate that cuproptosis-mediated upregulation of PD-L1 expression significantly enhances tumor sensitivity to αPD-L1 antibody therapy, providing robust evidence to support the potential clinical application of combining cuproptosis induction with immune checkpoint blockade strategies.

Moreover, Li et al. developed a PEG@Cu_2_O-ES nanocomposite capable of releasing a substantial amount of copper ions in tumor cells to induce cuproptosis, while near-infrared II (NIR-II) radiation-induced photothermal therapy (PTT) further accelerated copper ion release 66. Western blot analysis revealed a marked upregulation of PD-L1 protein expression in the PEG@Cu₂O-ES + NIR-II treatment group. In a breast cancer mouse model, combination therapy with PEG@Cu_2_O-ES and anti-programmed death-1 (αPD-1) antibody resulted in minimal tumor volume and weight, accompanied by reduced Ki-67 expression. In another study, Yan et al. engineered an inhalable nanoparticle platform (OMP) to accumulate efficiently in pulmonary tissues and induce cuproptosis 22. Flow cytometry and ELISA analyses demonstrated concurrent increases in both membrane-bound PD-L1 and soluble PD-L1 levels following OMP treatment in B16F10 cells. In a B16F10 melanoma lung metastasis model, combined therapy with OMP and αPD-L1 antibody significantly expanded the proportion of tumor-infiltrating CD8⁺ T cells and promoted M1 macrophage polarization. Notably, this therapeutic strategy also reduced immunosuppressive populations, including Tregs and MDSCs, thereby mitigating the immunosuppressive TME and augmenting antitumor immune responses.

Collectively, these studies highlight a novel paradigm in which engineered nanomaterials induce cuproptosis to upregulate PD-L1 expression, creating a favorable landscape for enhanced immune checkpoint inhibitor therapy. This innovative approach represents a significant advancement in the field of cancer immunotherapy, offering promising translational potential for precision medicine applications.

### 2.4. Activation of the cGAS-STING pathway

Recent studies have demonstrated that cuproptosis can activate the cGAS-STING signaling axis by releasing mtDNA, thereby triggering a potent antitumor immune response 21. During this process, excessive copper ions accumulate in the mitochondria, directly binding to lipoylated enzymes in the TCA cycle. This interaction induces the irreversible aggregation of lipoylated proteins and destabilization of Fe-S cluster proteins, leading to severe proteotoxic stress in the mitochondria and disruption of the respiratory chain. The increased permeability of the damaged mitochondrial membrane allows mtDNA to leak into the cytosol, where it is detected by the DNA sensor cGAS 67. Activated cGAS catalyzes the synthesis of the second messenger cyclic GMP-AMP, which subsequently binds to the adaptor protein STING. This initiates the activation of TANK-binding kinase 1, followed by the phosphorylation of interferon regulatory factor 3 and activation of nuclear factor kappa-light-chain-enhancer of activated B cells (NF-κB) 68. These molecular events collectively enhance the secretion of type I interferons, such as interferon-β and pro-inflammatory cytokines 69. The release of these immune mediators enhances the maturation of DCs, augmenting their antigen-presenting capabilities. Additionally, the increased cytokine production promotes the infiltration of cytotoxic CD8^+^ T cells into tumor tissues. This coordinated immune response results in a potent and sustained antitumor immunity, highlighting the critical role of cuproptosis-mediated cGAS-STING axis activation in cancer immunotherapy 70.

Jiang et al. established a co-culture system of DCs and renal carcinoma cells, demonstrating that tumor cell cuproptosis significantly elevated intracellular cGAMP levels in DCs 71. Furthermore, the expression of key molecules in the cGAS-STING signaling pathway was upregulated at both the protein and mRNA levels in a dose-dependent manner. In tumor-bearing mouse models, inoculation with renal carcinoma cells pretreated with a cuproptosis inducer resulted in a significantly higher proportion of CD45⁺CD8⁺ T lymphocytes in peripheral blood compared to the control group. These findings underscore the role of cuproptosis in enhancing antitumor immune responses through the activation of the cGAS-STING pathway. To address the therapeutic challenges associated with TNBC, Xu et al. developed a novel nanodrug, CGNPs, which exhibits tumor-specific accumulation *via* a dual-targeting mechanism: passive targeting through the EPR effect and active transmembrane transport mediated by the tumor-penetrating peptide (tLyp1) 72. The nanocarrier delivers glucose oxidase (GOx) to the tumor, which induces intracellular acidification. This pH reduction triggers a smart-responsive disassembly of the CGNPs framework, leading to the complete release of copper ions and the induction of cuproptosis. Consequently, this process generates significant proteotoxic stress and promotes leakage of mtDNA into the cytosol. The released mtDNA activates the cGAS-STING signaling axis, triggering a cascade of immune responses that effectively reverse the immunosuppressive TME, resulting in significant inhibition of tumor growth and metastasis. In another study, Liu et al. synthesized hyaluronic acid (HA)-modified zinc-copper bimetallic peroxide (ZCPO@HA) nanoparticles using a one-step symbiotic method 73. These nanoparticles were employed to induce cuproptosis and activate the immune system *via* the cGAS-STING pathway. Transmission electron microscopy observations revealed that 4T1 cells treated with ZCPO@HA nanoparticles exhibited disrupted cellular structures and aggravated mitochondrial damage. Polymerase chain reaction analysis demonstrated significant release of mtDNA from treated cells. Western blot confirmed robust expression of phosphorylated TANK-binding kinase 1 and phosphorylated interferon regulatory factor 3, leading to the secretion of interferon-β and activation of the cGAS-STING signaling pathway.

### 3. Nanoplatforms for cuproptosis induction

Recent advancements in nanotechnology have led to the development of diverse copper-based nanomaterials to modulate intracellular copper levels and effectively trigger cuproptosis. These nanomaterials include copper ionophore-based nanoparticles, copper-based metal-organic frameworks (Cu-MOFs), copper-based inorganic nanomaterials, copper-based organic nanomaterials, and copper-based hybrid nanomaterials, each engineered to leverage specific physicochemical properties for precise Cu^2+^ delivery and release. By responding to the distinct conditions of the TME, these nanomaterials enhance oxidative stress, disrupt cellular homeostasis, and offer targeted therapeutic potential. Despite their promise, challenges such as limited delivery efficiency, copper leakage, and unclear degradation pathways persist, necessitating further research to optimize these platforms for clinical translation.

### 3.1. Copper ionophore-based nanoparticles

Copper ionophores, such as ES and DSF, chelate copper ions (Cu^2+^) to facilitate their mitochondrial delivery, thereby initiating cuproptosis through mitochondrial proteotoxic stress. Lu et al. constructed an ES@CuO nanoplatform by encapsulating ES and CuO nanoparticles in a PEG polymer shell 74. This system exhibits pH-responsive behavior, releasing ES and Cu^2+^ in the acidic TME. The released complex subsequently targets mitochondria, inducing the aggregation of lipoylated proteins such as DLAT, thereby initiating cuproptosis. In another study, Zhao et al. engineered DSF-loaded copper sulfide nanoparticles templated by CpG oligonucleotides (DSF/CuS-C), leveraging the Toll-like receptor agonist CpG as a structural motif 28. Upon photothermal activation, these nanoparticles efficiently initiated cuproptosis and subsequently elicited a robust antitumor immune response. These studies highlight the significant therapeutic potential of copper ionophore-based nanoparticles in cancer treatment. However, challenges remain, including their limited tumor selectivity and the risk of systemic copper homeostasis disruption, which must be addressed to advance clinical translation.

### 3.2. Cu-MOFs

MOFs are synthesized through the self-assembly of transition metal ions and organic ligands. Their structural versatility, high cargo capacity, and responsiveness to the TME make them highly valuable for applications in catalysis and nanomedicine 75. Among these materials, Cu-MOFs have garnered significant attention due to their unique ability to induce cuproptosis, presenting promising opportunities for advancing cancer therapy. For instance, Yi et al. developed FA-PZ@MOF NPs capable of co-delivering copper ions and pyroptosis-inducing agents 76. These nanoparticles demonstrated exceptional stability, tumor-targeting capability, and the ability to simultaneously induce cuproptosis and pyroptosis. Such dual-modality effects collectively enhanced ICD promoted DC maturation, and increased CD8^+^ T-cell infiltration in hepatocellular carcinoma models. In another study, Li et al. engineered Cu-BTC@DDTC incorporating the DSF metabolite 77. These frameworks exhibited high drug-loading efficiency, stability, and potent antitumor activity, especially when combined with low-dose chemotherapy. Despite these advancements, further optimization of MOFs is necessary to address key challenges, including improving biodegradability, incorporating biocompatible linkers, and simplifying synthesis protocols, all of which are critical for enhancing their clinical translational potential 78.

### 3.3. Copper-based inorganic nanomaterials

Owing to their unique nanoscale dimensions, large surface areas, and distinctive architectural features, inorganic nanomaterials exhibit remarkable biological behaviors, including tumor-responsive imaging capabilities, sensitivity to external stimuli, and intrinsic enzyme-mimetic activities. These properties make them highly promising for applications in cancer theranostics 79. Chen et al. developed a novel Cu_2_O@Mn_3_Cu_3_O_8_ (CMCO) core-shell nanozyme that leverages photothermal-enhanced catalytic mimicry to potentiate ferroptosis-driven cuproptosis, specifically targeting colorectal cancer 80. This innovative design enables precise modulation of the TME, inducing efficient ferroptosis-driven cuproptosis while maintaining excellent *in vivo* biosafety and tumor-suppressive efficacy. In another study, Song et al. engineered multifunctional CuSiO_3_@Au-Pd nanomotors by integrating Au-Pd nanoalloys onto self-assembled flower-like CuSiO_3_ structures 81. This asymmetric design not only enhances catalytic properties but also imparts photothermal-enhanced Fenton-like activity and autonomous mobility, significantly amplifying oxidative stress levels to induce cuproptosis. Despite their therapeutic potential, the long-term toxicity of metal ions during metabolic processes remains a concern. To address this, copper-based inorganic nanomaterials should be rationally designed to integrate advanced functionalities such as imaging capabilities, biocompatibility, and biodegradability. Such designs enable precise monitoring of their *in vivo* biodistribution, dynamic degradation profiles, and metabolic fate, ensuring safer and more effective therapeutic outcomes 82.

### 3.4. Copper-based organic nanomaterials

Organic polymers, rich in peripheral functional groups and exhibiting excellent biocompatibility and drug-loading capacities, have emerged as highly promising nanoplatforms for enhancing cuproptosis-based tumor therapy 17. Xu et al. designed a multifunctional CuET-doped, artemisinin (ART)-loaded hollow nanoplatform (ART@CuT/ETH HNP) *via* a chelation competition-driven hollowing strategy 83. This innovative design incorporated surface modifications and both coordination and disulfide bonds, enabling rapid disassembly in the acidic and GSH-rich TME. This process effectively triggered the release of Cu^2+^ ions and ART. Notably, the abundant disulfide bonds in the 3,3'-dithiobis(propionohydrazide) (TPH) framework further depleted intracellular GSH levels, amplifying oxidative stress and consequently promoting cuproptosis. Building on this success, Xu et al. subsequently engineered a TPH-modified hyaluronan-functionalized hollow Ca/Cu bimetallic nanoplatform (D@HCC-CuTH) 84. This advanced platform was specifically designed to intensify endoplasmic reticulum stress and disrupt mitochondrial function, thereby inducing robust cuproptotic activity in tumor tissues and achieving remarkable inhibition of tumor progression. While these platforms exemplify rational functionalization and precise payload control, challenges persist regarding limited copper capacity and potential copper leakage. Future research should focus on enhancing structural stability and refining release mechanisms to address these limitations.

### 3.5. Copper-based hybrid nanomaterials

Organic-inorganic hybrid nanomaterials, which combine the physicochemical properties of polymer matrices with the inherent advantages of inorganic nanoparticles, offer complementary benefits and synergistic effects. This unique combination has led to growing interest in copper-based hybrids for cuproptosis-driven cancer therapy. For instance, Yang et al. developed Au NCs-Cu^2+^@SA-HA hybrid nanogels that exhibited excellent biocompatibility and selective CD44 receptor recognition 85. These nanogels enabled NIR-responsive imaging and TME-triggered Cu^2+^ release, effectively inducing synergistic cuproptosis. Similarly, Zhang et al. engineered a copper single-atom nanozyme (Cu SAzyme) embedded in a mesoporous organosilicon framework capable of stably adsorbing both Cu and phloretin 86. Upon endogenous GSH-triggered degradation, Cu ions are released to induce cuproptosis, whereas unreleased Cu atoms transform into atomic Cu-O centers to form SAzymes that facilitate photothermally enhanced ROS generation. In combination with phloretin-mediated glycolysis disruption, this *in situ*-activated system synergistically promotes cuproptosis while minimizing off-target effects. Copper-based hybrid nanomaterials hold significant potential for imaging-guided therapy and controlled release applications; however, their clinical translation is currently hindered by challenges such as low tumor delivery efficiency, difficulties in precisely regulating Cu^2+^ release, and limited understanding of degradation and clearance pathways 87. To address these limitations, a promising strategy involves designing streamlined, biodegradable, and traceable hybrid platforms combined with companion imaging techniques. These systems would enable real-time verification of intratumoral delivery, thereby enhancing safety, supporting personalized treatment approaches, and facilitating regulatory acceptance.

## 4. Nanoparticles that induce cuproptosis enhance the antitumor efficacy of immunotherapy

### 4.1. Cuproptosis monotherapy

Cuproptosis exhibits distinct advantages in enhancing antitumor immunotherapy. Although conventional chemotherapy or RT directly kill tumor cells, they also induce significant alterations in the TME that promote therapeutic resistance 88. These changes enable tumor cells to secrete a variety of immunosuppressive cytokines, which not only suppress the function of immune cells but also enhance their own proliferative, migratory, and invasive capabilities, ultimately resulting in distant metastasis and tumor recurrence 89. Furthermore, apoptosis triggered by chemotherapy and RT hinders the release of tumor-associated antigens (TAAs) and DAMPs from dying cancer cells into the extracellular milieu, thereby compromising immune system activation. In contrast, cuproptosis offers a superior strategy for cancer treatment. This specialized form of cell death not only eradicates tumor cells through copper-dependent cytotoxicity but also elicits ICD, which facilitates the extensive release of TAAs and DAMPs, thereby activating systemic antitumor immunity. Consequently, cuproptosis not only achieves effective primary tumor elimination but also exerts robust suppression on distant metastasis and tumor recurrence, representing a promising approach for overcoming the limitations of conventional cancer therapies 74.

Recent studies have identified that cuproptosis mediated by copper complexes, such as bis (diethyl dithiocarbamate) copper (CuET), is a promising cancer treatment strategy 90. Lu et al. developed a novel TPP-modified CuET derivative, termed TPP-CuET, for cuproptosis-based cancer therapy 39. *In vitro* experiments showed that TPP-CuET induced a substantially higher mitochondrial copper accumulation in 4T1 breast cancer cells compared to both the CuET-treated group (197.3% increase) and the untreated group (16.5-fold higher), thereby triggering a cascade of cuproptotic reactions. The tumor-targeting ability of TPP-CuET is primarily attributable to the TPP moiety, which preferentially accumulates within the mitochondria of cancer cells as a result of their elevated mitochondrial membrane potential, thereby enabling selective copper delivery and amplifying cuproptosis. Immunogenic analysis revealed that TPP-CuET treatment promoted the release of DAMPs, including CRT exposure, ATP, and HMGB1. To evaluate the immunomodulatory effects, conditioned medium from DAMP-treated 4T1 cells was administered to RAW 264.7 macrophages. Flow cytometry analysis confirmed that TPP-CuET-treated conditioned medium significantly augmented the ratio of CD86^+^ M1 macrophages compared to the control group. Further transcriptome sequencing revealed that TPP-CuET activated the expression of genes related to the MHC-I antigen presentation pathway, thereby promoting CD8^+^ T cell and NK cell activation. These findings underscore the therapeutic potential of TPP-CuET as a novel strategy to induce cuproptosis and stimulate anti-cancer immunity.

To achieve the effective aggregation and precise release of copper ions in tumor cells, a variety of stimulus-responsive nanomaterials have been applied to trigger cuproptosis. Chang et al. synthesized PDA-diethyldithiocarbamate/copper (PDA-DTC/Cu) nanoparticles under alkaline conditions, with PDA serving as a chelating agent for copper ions and diethyldithiocarbamate acting as a specific ligand for copper binding 47. Physicochemical characterization revealed that PDA-DTC/Cu NPs exhibited an average hydrodynamic diameter of 150 nm and demonstrated acid-triggered release behavior, underscoring their potential for tumor-targeted delivery. *In vivo* studies demonstrated that PDA-DTC/Cu nanoparticles significantly inhibited tumor growth in 4T1 tumor-bearing mice, achieving an impressive tumor inhibition rate of 66.3%. Notably, these nanoparticles also enhanced immune cell infiltration into the TME and substantially reduced the population of MDSCs, thereby remodeling the immunosuppressive TME. All experiments were performed with a minimum of three independent replicates. Statistical analyses were conducted in GraphPad Prism, employing unpaired Student's t-tests for comparisons between two groups and one-way ANOVA for multiple comparisons. These findings highlight the dual functionality of PDA-DTC/Cu nanoparticles in effectively inducing cuproptosis and simultaneously modulating the immunosuppressive TME, providing a promising therapeutic strategy for cancer treatment.

In a recent study, Hu et al. synthesized a dinitrogen-diphosphine chelated copper complex (Cu(I)) using one-pot synthesis approach and subsequently self-assembled it with ROS-sensitive polymers to form Cu(I) nanoparticles (Cu(I) NPs) 25. After co-incubation for 36 h, the copper release rate of Cu(I) NPs in hydrogen peroxide (H_2_O_2_) solution reached 91.4%, which was significantly higher than that in PBS (11.5%). Atomic absorption spectrometry revealed that the intracellular copper accumulation in tumor cells treated with Cu(I) NP was 4-fold higher than that in tumor cells treated with copper chloride, demonstrating efficient cellular uptake. In the Panc02 pancreatic cancer mouse model, the tumor volume in mice treated with Cu(I) NP was significantly inhibited, with the average tumor mass measuring only 174 mg, significantly lower than that of the control group. Flow cytometry analysis showed that Cu(I) NP treatment effectively increased the proportion of mature DCs in tumors compared to oxaliplatin treatment. Additionally, the intra-tumoral infiltration of CD8^+^ T cells of the Cu(I) NP treatment group was 1.5 times that of the oxaliplatin treatment group. Cu(I) NP also reprogramed macrophages from M2 type to M1 type. The ratio of M1 to M2 macrophage in the Cu(I) NP treatment group was approximately 1.6, which was five times higher than that in the oxaliplatin treatment group. Therefore, stimulus-responsive copper complex nanoparticles present a viable strategy for inducing cuproptosis and enhancing cancer immunotherapy.

**Figure 1:**
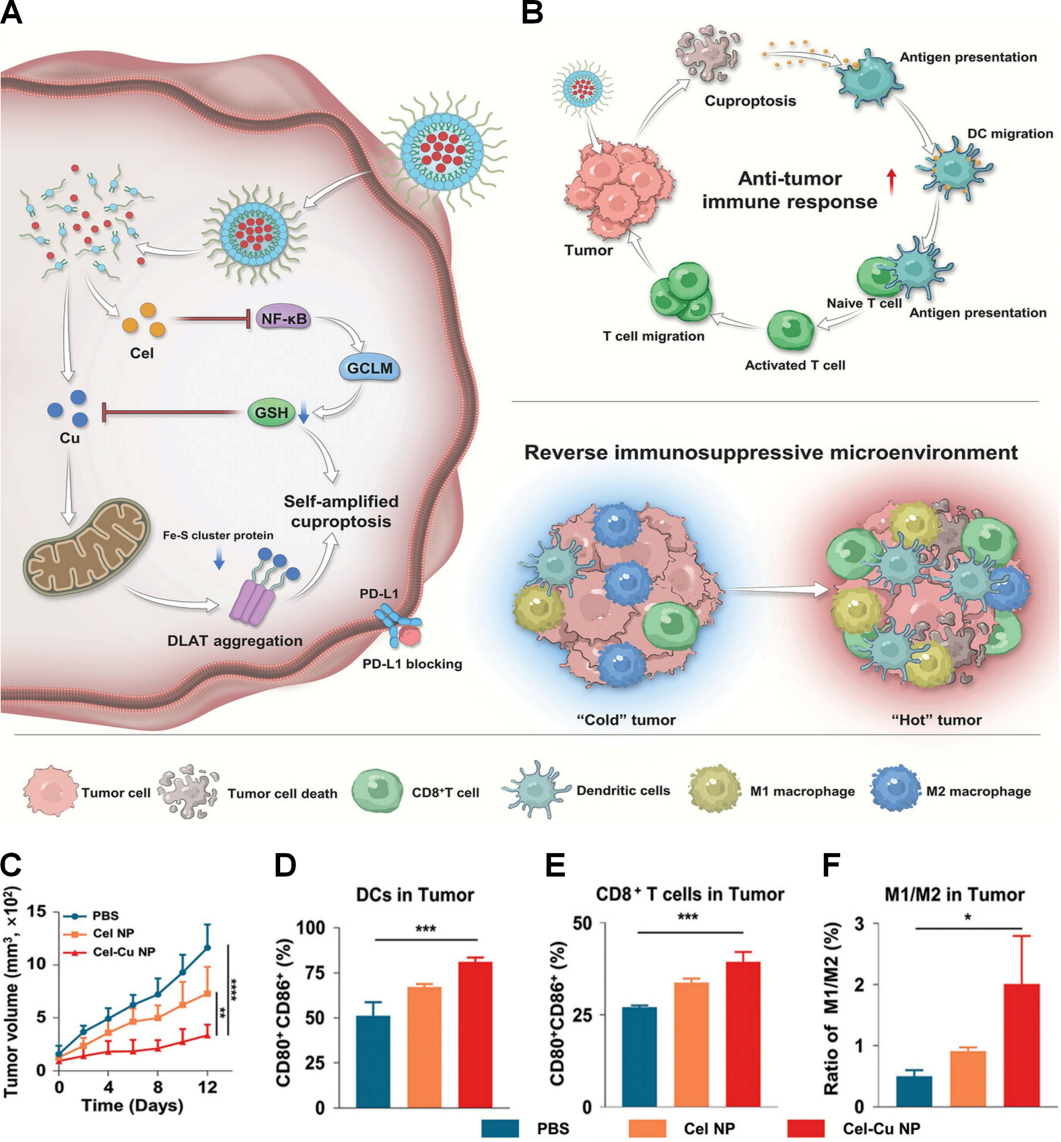
Cel-Cu NPs amplify cuproptosis through GSH depletion and enhance antitumor immunotherapy efficacy. (A) The biological mechanism underlying the self-reinforcing cuproptosis induced by Cel-Cu NPs. (B) The mechanism by which Cel-Cu NPs trigger antitumor immune responses. (C) Tumor growth inhibition curves in 4T1 tumor-bearing mice across different treatment groups. (D) Proportion of mature DCs in the tumor tissues of 4T1 tumor-bearing mice across different treatment groups. (E) Proportion of CD8^+^ T cells in the tumor tissues of 4T1 tumor-bearing mice across different treatment groups. (F) Quantification of the M1/M2 macrophage ratio in the tumor tissues of 4T1 tumor-bearing mice across different treatment groups. *P < 0.05, **P < 0.01, ***P < 0.001, ****P < 0.0001. Reproduced with permission from Wiley-VCH (journal citation: 92).

Elevated intracellular GSH in tumor cells acts as a critical barrier to cuproptosis by directly chelating copper ions, forming inert Cu(I)-GSH complexes that prevent copper-dependent proteotoxic stress 91. To address this issue, Lu et al. fabricated GSH-scavenging celastrol-copper nanoparticles (Cel-Cu NPs) to self-amplify cuproptosis and enhance immunotherapy (Figures 1A and B) 92. Cel was coordinated with copper ions to prepare Cel-Cu complex, which was further encapsulated in DSPE-PEG_2000_ to obtain Cel-Cu NPs. *In vitro* experiments confirmed that Cel-Cu NPs successfully delivered copper ions into tumor cells. GSH detection showed that Cel-Cu NPs reduced the GSH level in 4T1 cells by 53%, which was attributed to the inhibition of the NF-κB pathway by Cel. Further experiments showed that Cel-Cu NPs induced intense cuproptosis in 4T1 cells. In 4T1 tumor-bearing mouse model, Cel-Cu NPs achieved the best tumor suppression effect (Figure 1C). Flow cytometry analysis showed that the proportion of mature DCs in the tumor tissues of the Cel-Cu NPs treatment group was 1.6 times higher than that of the PBS treatment group (Figure 1D). Compared with PBS and Cel NPs, Cel-Cu NPs upregulated the proportion of CD8^+^ T cells in tumor tissues by 1.5-fold and 1.2-fold, respectively (Figure 1E). In addition, the M1/M2 macrophage ratio of the Cel-Cu NPs treatment group was four times higher than that of the PBS treatment group (Figure 1F). These findings suggest that Cel-Cu NPs induces cuproptosis and triggers a robust antitumor immune response.

### 4.2. Cuproptosis combined with chemotherapy

Drug resistance represents a critical challenge in chemotherapy, primarily mediated by the aberrant TME characterized by hypoxia and elevated GSH levels. Hypoxia stabilizes hypoxia-inducible factor-1α, leading to upregulation of glucose transporters and multidrug resistance protein 2 that promote chemotherapeutic drug efflux 65, while excessive GSH directly inactivates anticancer drugs and facilitates their MRP2-mediated extrusion. Cuproptosis presents a promising strategy to overcome these resistances. The accumulated copper ions in cuproptosis participate in Fenton-like reactions that generate ROS, effectively downregulating hypoxia-inducible factor-1α-mediated drug efflux pumps 93. Simultaneously, copper ions deplete intracellular GSH by forming Cu(I)-GSH complexes, thereby preventing GSH-mediated drug detoxification and restoring chemotherapeutic efficacy 94. This dual function of cuproptosis disrupts two major pillars of chemoresistance. Importantly, chemotherapeutic agents reciprocally enhance tumor cell susceptibility to cuproptosis by inducing endoplasmic reticulum stress and mitochondrial dysfunction, establishing a self-amplifying therapeutic cycle 95. This synergistic interplay between cuproptosis and chemotherapy not only overcomes conventional resistance mechanisms but also remodels the immunosuppressive TME, thereby creating a favorable milieu for enhanced antitumor immunity 96. The complementary mechanisms of these two modalities offer a transformative strategy for improving therapeutic outcomes of refractory malignant tumors.

TNBC is the subtype of breast cancer with the poorest prognosis, and chemotherapy remains the main treatment strategy for advanced TNBC. Most chemotherapy drugs mainly exert antitumor effects by inducing apoptosis of tumor cells. However, tumor cells usually evade apoptosis through multiple mechanisms, thereby leading to treatment resistance. Combining apoptosis with other forms of programmed cell death may help overcome chemotherapy resistance. Wang et al. developed multifunctional nanoparticle PCD@Cu composed of PEG-thioketal-doxorubicin (DOX), PEG-diethylenetriaminepentaacetic acid-disulfide-camptothecin, and Cu^2+^ for the synergistic therapy of breast cancer 97. *In vitro* experiments showed that PCD@Cu exhibited excellent ROS and GSH-responsive drug release behaviors and was effectively taken up by 4T1 cells. RNA sequencing revealed that PCD@Cu upregulated the genes related to copper entry and downregulated the genes responsible for copper excretion, indicating that PCD@Cu increased the accumulation of copper in 4T1 cells. In 4T1 tumor-bearing mouse model, the tumor inhibition rate in the PCD@Cu treatment group reached approximately 80.7%, obviously higher than that in the other groups. Quantitative real-time polymerase chain reaction analysis showed that PCD@Cu significantly upregulated the expression of Cgas, CCL5, and CXCL10 in tumor tissues, indicating that PCD@Cu activated the cGAS/STING pathway. Flow cytometry and immunohistochemical analysis demonstrated that PCD@Cu markedly promoted the maturation of DCs, increased the infiltration of CD8⁺ T cells, and upregulated the expression of granzyme B. Collectively, PCD@Cu exhibited remarkable antitumor activity, underscoring its potential as a safe and effective nanodrug delivery platform for the treatment of TNBC.

Immunotherapy combined with chemotherapy has shown improved outcomes in advanced TNBC. However, combination therapy often yields limited clinical response rates and suboptimal outcomes due to the innate or acquired therapeutic resistance of cancer cells and the immunosuppressive nature of the TME 34. Therefore, there is an urgent need to develop innovative strategies that can effectively overcome chemotherapy resistance and remodel the TME. Zhang et al. synthesized dynamic nanoparticles (GPCuD NPs) self-assembled from DOX prodrug and Cu^2+^ to induce cuproptosis and enhance the combined chemotherapy-immunotherapy for breast cancer 98. Dynamic light scattering (DLS) detection shows that the size of GPCuD NPs was 166 nm ± 0.2 nm. Matrix metalloproteinase-2-cleavable peptide and pH-reversible phenylboronate ester bond were included in GPCuD NPs to ensure their effective tumor accumulation and deep penetration. Once GPCuD NPs were internalized by tumor cells, the overexpressed GSH and the acidic conditions in tumor cells triggered the release of Cu^2+^ and DOX prodrug from GPCuD NPs, thereby inducing cuproptosis and apoptosis. Western blot analysis showed that GPCu NPs downregulated the expression of FDX1, LIAS, and DLAT in 4T1 cells, confirming that they successfully triggered cuproptosis. In 4T1 tumor-bearing mice, the GPCuD NPs treatment group exhibited the smallest tumor volume and tumor weight. Flow cytometry showed that the proportion of mature DCs in GPCuD NPs treatment group was 3.4-fold and 1.7-fold that of the control group and the CuCl_2_ treatment group, respectively. Compared with the control group, the levels of CD4^+^, CD8^+^ T cells, and CTLs in the tumors of GPCuD NPs treatment group were substantially increased. Furthermore, GPCuD NPs effectively reduced the infiltration of Tregs cells in the tumor. All experiments were performed in biological triplicates (n = 3) and analyzed using one-way ANOVA/Tukey. GPCuD NPs are expected to solve the current predicament of low effective rate in TNBC treatment. Nevertheless, additional cross-validation experiments conducted across different laboratories and model systems are essential to further confirm the translational relevance of these results.

### 4.3. Cuproptosis combined with RT

RT, a cornerstone in cancer treatment, kills tumor cell by directly damaging DNA through high-energy ionizing radiation or indirectly generating ROS. While offering distinct advantages including non-invasiveness, precise tumor targeting, and broad clinical applicability, RT efficacy is frequently hindered by the hypoxic nature of the TME, elevated antioxidant defenses mediated by high GSH levels, and acquired radioresistance, which collectively contribute to dose-limiting normal tissue toxicity and increased risk of tumor recurrence 99. Cuproptosis presents promising opportunities to overcome these limitations. It exhibits remarkable synergy with RT-induced metabolic reprogramming and oxidative stress, wherein copper ions perpetually deplete GSH through Fenton-like reactions, simultaneously relieving tumor hypoxia and shifting tumor glycolytic metabolism toward oxidative phosphorylation, thereby substantially enhancing radiosensitivity 100. Furthermore, the combination of cuproptosis and RT demonstrates potent immunomodulatory effects, cooperatively inducing ICD and reprogramming the immunosuppressive TME.

The integration of nanomaterials in RT and copper-mediated cancer cell death offers enhanced precision and therapeutic synergy, enabling targeted delivery and amplifying oxidative damage in tumors 101. Nanomaterials can modulate the release of copper ions and radiation-induced stress, thus maximizing antitumor efficacy while minimizing off-target effects. Li et al. engineered a multifunctional copper-based nanocomposite, RCL@Pd@CuZ, as a cuproptosis inducer to boost radioimmunotherapy 102. Specifically, ultrasmall palladium nanozyme-loaded Cu MOF was encapsulated in DSPE-PEG-RGD-modified and capsaicin-embedded liposomes to prepare RCL@Pd@CuZ lipidosome. The incorporation of the RGD motif enables specific binding to the integrin α_v_β_3_ receptor, a hallmark of tumor cells, thereby ensuring precise and efficient tumor accumulation through active targeting. Once RCL@Pd@CuZ is taken up by tumor cells, Cu^2+^ is released to induce cuproptosis, thereby generating hydroxyl radicals (•OH) and eliminating GSH. Meanwhile, ultrasmall palladium nanozyme decomposes H_2_O_2_ to produce ROS and oxygen, which, together with capsaicin, improve tumor hypoxia. In addition, Pd promotes the deposition of X-rays to synergistically sensitize RT. *In vitro* studies demonstrated that RCL@Pd@CuZ significantly reduced GSH levels in tumor cells and inhibited mitochondrial respiration, potentiating RT-induced cell death *via* cuproptosis and ferroptosis in MC38 cells. In C57BL/6 tumor-bearing mice, the tumor volume in the RCL@Pd@CuZ + RT group was only 3.6% of that in the RT group. Compared with RT alone, RCL@Pd@CuZ + RT significantly increased the levels of CRT and HMGB1 in the tumor, confirming the occurrence of a more intense ICD. In tumor-draining lymph nodes (TDLNs), the proportion of CD86^+^CD11c^+^ DCs in the RCL@Pd@CuZ + RT group was 59.24% ± 1.21%, which was markedly higher than that in the RT group (43.14% ± 1.63%). The therapeutic efficacy was further augmented when combining RCL@Pd@CuZ + RT with αPD-1 therapy, achieving maximal tumor growth suppression through increased T lymphocyte infiltration and activation. This study underscores the potential of nanomaterial-mediated cuproptosis as a powerful strategy to enhance RT and immunotherapy, offering new avenues for cancer treatment.

RT-induce ICD enhances antitumor immunity by releasing tumor antigens and recruiting immune cells. However, its application is limited by off-target effects, immunosuppressive TME, and low tumor immunogenicity, which may reduce therapeutic efficacy. Cuproptosis can amplify RT-induced ICD by disrupting mitochondrial metabolism, increasing oxidative stress, and promoting DAMP release. Jiang et al. fabricated a bimetallic hybrid nanostimulator (CHP) composed of Cu^2+^ and Hf^4+^-doped phosphate backbone and poly(vinylpyrrolidone) for radio-cuproptosis-immunotherapy of TNBC 103. In the acidic TME, Cu^2+^ and Hf^4+^ are released from CHP. Cu^2+^ sensitizes RT by consuming GSH, generating oxygen, increasing oxidative stress, and triggering cuproptosis. Meanwhile, Hf^4+^, as a high atomic-number (Z) element, enhances the antitumor efficacy of RT by promoting dose deposition. *In vitro* experiments confirmed that CHP significantly reduced the GSH level in tumor cells and increased the levels of ROS and oxygen, thereby creating a TME conducive to RT. Subsequent studies showed that CHP triggered cuproptosis in tumor cells, which was further enhanced by X-ray irradiation. In a bilateral 4T1 tumor model, the group receiving CHP + RT treatment demonstrated the most robust therapeutic efficacy, achieving tumor inhibition rates of 82.1% and 77.0% for primary and distant tumors, respectively. Flow cytometry analysis showed that CHP + RT increased the proportion of mature DCs in lymph nodes from 19.9% (control) to 49.0%. The proportion of CD4^+^ T cells and CD8^+^ T cells in the distant tumors of CHP + RT treatment group was 3.55 times and 3.71 times higher than that of the control group, respectively. These findings collectively demonstrate that low-dose X-rays combined with CHP effectively reprograms the immunosuppressive TME, eliciting a robust antitumor immune response with significant implications for cancer immunotherapy.

Since both cuproptosis and RT rely on an oxygen-rich TME, their efficacy is often compromised by tumor hypoxia 104. To address this limitation, Pei et al. engineered a nanosystem (^Cu/AP^H-M) for efficient copper ion delivery and tumor hypoxia reversal 101. Specifically, the fabrication of ^Cu/AP^H-M involved the deposition of gold-platinum (Au-Pt) bimetallic nanozymes onto hollow mesoporous Prussian blue nanoparticles, followed by Cu^2+^ doping and encapsulation within a tumor cell membrane. The hydrodynamic size and Zeta potential of ^Cu/AP^H-M were 115 nm and -34 mV, respectively. Under pH 5.6 conditions, the cumulative release of Cu^2+^ from ^Cu/AP^H-M reached 85.16% within 48 h. ^Cu/AP^H-M exhibited robust catalase-like activity, continuously decomposing H_2_O_2_ into oxygen while facilitating targeted release of Cu^2+^ to induce cuproptosis. In CT26 tumor-bearing mice, ^Cu/AP^H-M exhibited effective tumor accumulation and retention, which can be attributed to its membrane camouflage effect. Subsequent studies revealed that ^Cu/AP^H-M significantly inhibited the growth of subcutaneous solid tumors. Flow cytometry analysis further demonstrated that compared to the control group, the proportion of mature DCs in the lymph nodes of mice treated with ^Cu/AP^H-M + RT + αPD-L1 increased to 25.5 ± 2%. Additionally, following treatment with ^Cu/AP^H-M + 6 Gy radiation, the proportion of effector memory T cells in the spleen rose to 39.14 ± 1.86%. Notably, the addition of αPD-L1 further enhanced the effector memory T cell population to 48.6 ± 3.56%. All experiments were conducted with biological replicates (typically n = 3-6) and analyzed using one- or two-way ANOVA. These results suggest that the combination of ^Cu/AP^H-M with RT and αPD-L1 not only enhances antitumor immune responses but also induces durable immune memory, effectively preventing tumor recurrence. However, further rigorous validation across multiple laboratories and diverse tumor models is essential to strengthen the translational potential and clinical relevance of these findings.

### 4.4. Cuproptosis combined with ICIs

ICIs, such as antibodies targeting PD-1, PD-L1, CTLA-4, and T cell immunoreceptor with Ig and ITIM domains (TIGHT), reinvigorate antitumor T-cell responses through blockade of inhibitory signaling pathways. However, their clinical potential is often hindered by the immunosuppressive TME. Recent studies reveal that cuproptosis exhibits profound immunomodulatory properties. Specifically, cuproptosis elicits dual effects: on one hand, it upregulates PD-L1 expression in tumor cells, potentially enhancing their susceptibility to ICIs; on the other hand, it induces robust ICD, characterized by the release of DAMPs, such as HMGB1, ATP, and cytosolic DNA, which collectively promote DC maturation and CTL infiltration 105. The integration of cuproptosis-inducing agents with ICIs represents a highly synergistic therapeutic strategy 106, 107. While cuproptosis-mediated ICD initiates and enhances antitumor immunity by increasing tumor immunogenicity and promoting T-cell priming, ICIs act downstream to prevent T-cell exhaustion by preserving effector functions and maintaining durable immune responses 108. Consequently, this dual mechanism not only potentiates immediate tumor control but also establishes long-lasting immunological memory, as evidenced by persistent tumor-specific T-cell activity that effectively suppresses metastatic dissemination and prevents tumor recurrence 22. The convergence of these complementary mechanisms addresses the limitations of current immunotherapy approaches and provides a new approach to enhance cancer treatment efficacy 109.

The synergistic strategy of cuproptosis induction combined with ICIs presents a promising approach to overcome the limitations of ICI monotherapy. However, the high concentration of GSH in the TME can chelate copper ions, thereby compromising cuproptosis induction in tumor cells 110. To address this challenge, Wang et al. developed a platinum/copper dual-atom nanozyme (Pt/Cu DAzyme), in which Pt and Cu atoms, anchored on a poly(vinylpyrrolidone) scaffold, function as catalytic centers 106. The Pt/Cu DAzyme exhibits a remarkable ability to specifically enhance copper ion concentrations within tumors, thereby inducing cuproptosis. Moreover, the Pt component demonstrates GSH oxidase functionality, which reduces intracellular GSH levels in tumor cells, thus enhancing the effectiveness of cuproptosis. Both the Pt and Cu components also exhibit peroxidase activity, facilitating the conversion of H_2_O_2_ into •OH within the TME. This process results in mitochondrial dysfunction and ultimately leads to tumor cell death. The photothermal effect mediated by Pt further augments the therapeutic potential of the Pt/Cu DAzyme. Enzymatic assays revealed that Pt/Cu DAzyme significantly depleted intracellular GSH levels in CT26 cells through its dual-enzyme activity. Subsequent experiments demonstrated that Pt/Cu DAzyme induced hallmark features of cuproptosis in these cells. *In vivo* studies using CT26 tumor-bearing mice showed that Pt/Cu DAzyme + NIR irradiation + αPD-L1 achieved maximal inhibition of both primary tumor growth and distant metastatic progression. The percentage of mature DCs in the TDLNs of the combined treatment group was 40.50%, which was the highest among all groups. Notably, Pt/Cu DAzyme + NIR irradiation + αPD-L1 obviously reduced the level of MDSC in distant tumors and increased the proportion of intratumoral CD8^+^ T cells. Additionally, in a colorectal cancer liver metastasis model, the combination of αPD-L1 and Pt/Cu DAzyme markedly diminished‌ the number and size of hepatic metastatic lesions. These findings suggest that the combined therapeutic strategy enhances immune activation and T-cell responses.

**Figure 2:**
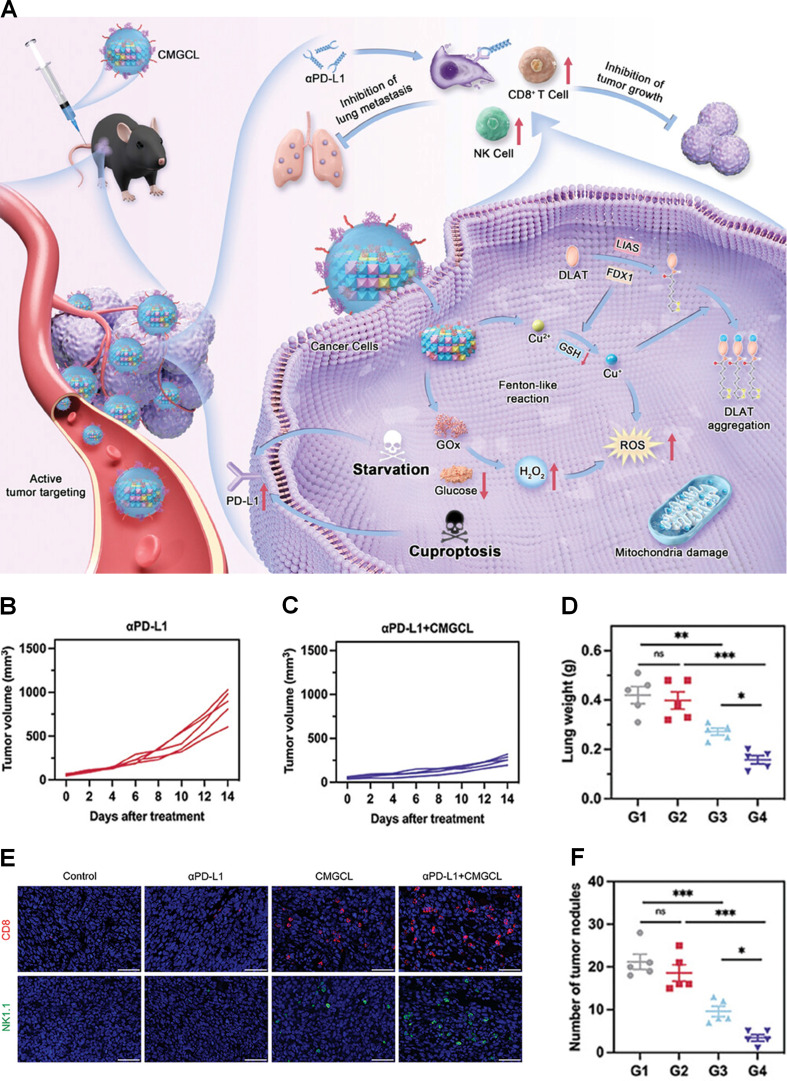
CMGCL potentiates the efficacy of antitumor immunotherapy by inducing cuproptosis and upregulating PD-L1 expression. (A) Schematic illustration of the mechanism by which CMGCL augments antitumor immune responses. Tumor volume curves in LLC tumor-bearing mice treated with αPD-L1 (B) and CMGCL + αPD-L1 (C). (D) Lung weights measured on day 14 across the different treatment groups in LLC tumor-bearing mice (n = 5). (E) Representative immunofluorescence images alongside quantification of relative fluorescence intensities of CD8 (red) and NK1.1 (green) in tumor sections from LLC tumor-bearing mice receiving different treatments (n = 3). Nuclei were counterstained with DAPI (blue). Scale bar: 50 µm. (F) Quantification of pulmonary metastatic nodules in LLC tumor-bearing mice receiving different treatments on day 14 (n = 5). *P < 0.05, **P < 0.01, ***P < 0.001, and ns indicates no significant difference. Reproduced with permission from Wiley-VCH (journal citation: 112).

Although αPD-L1 has shown promising therapeutic efficacy in the majority of lung cancer patients, its clinical benefit is often limited by low PD-L1 expression in the TME. Recent studies have shown that glucose starvation synergistically enhances cuproptosis-mediated upregulation of PD-L1 in lung cancer cells, thereby increasing their susceptibility to αPD-L1 therapy 111. Xu et al. engineered an innovative biomimetic nanomedicine (CMGCL) by encapsulating GOx-loaded copper-layered double hydroxide nanoparticles in lung cancer cell membranes to boost αPD-L1 therapy (Figure 2A) 112. The hydrodynamic size of CMGCL was approximately 93.9 nm, as detected by DLS. Under acidic conditions (pH 5.6), the cumulative release of Cu^2+^ from CMGCL over 24 h was significantly higher than that under neutral conditions (pH 7.4), confirming its excellent acid-responsive drug release. *In vitro* experiments demonstrated that CMGCL significantly downregulated the expression of FDX1 and LIAS and promoted the oligomerization of DLAT in lung cancer cells, effectively inducing cuproptosis. Furthermore, Western blot analysis revealed a marked upregulation of PD-L1 expression on these cells following CMGCL treatment. *In vivo* studies highlighted the efficient tumor-targeting capability of CMGCL, which may be attributed to its cancer cell membrane-based camouflage. In a Lewis lung carcinoma (LLC) tumor-bearing mouse model, combination therapy with CMGCL and αPD-L1 exhibited significantly enhanced tumor growth suppression compared to αPD-L1 monotherapy (Figures 2B and C). Flow cytometry analysis demonstrated the highest infiltration of CD8^+^ T cells and NK1.1^+^ cells in the combination treatment group (Figure 2E). Moreover, in a lung metastasis model, this combined treatment led to a significant reduction in both the number and size of metastatic nodules, resulting in decreased lung weights to 37.6% and 39.7% of those observed in the control and αPD-L1 groups, respectively (Figures 2D and F). All experiments were conducted with biological replicates (typically n = 3-5) and analyzed using ANOVA. While these results are encouraging, they remain preliminary and require further validation through independent confirmation and testing across additional diverse preclinical models.

**Figure 3:**
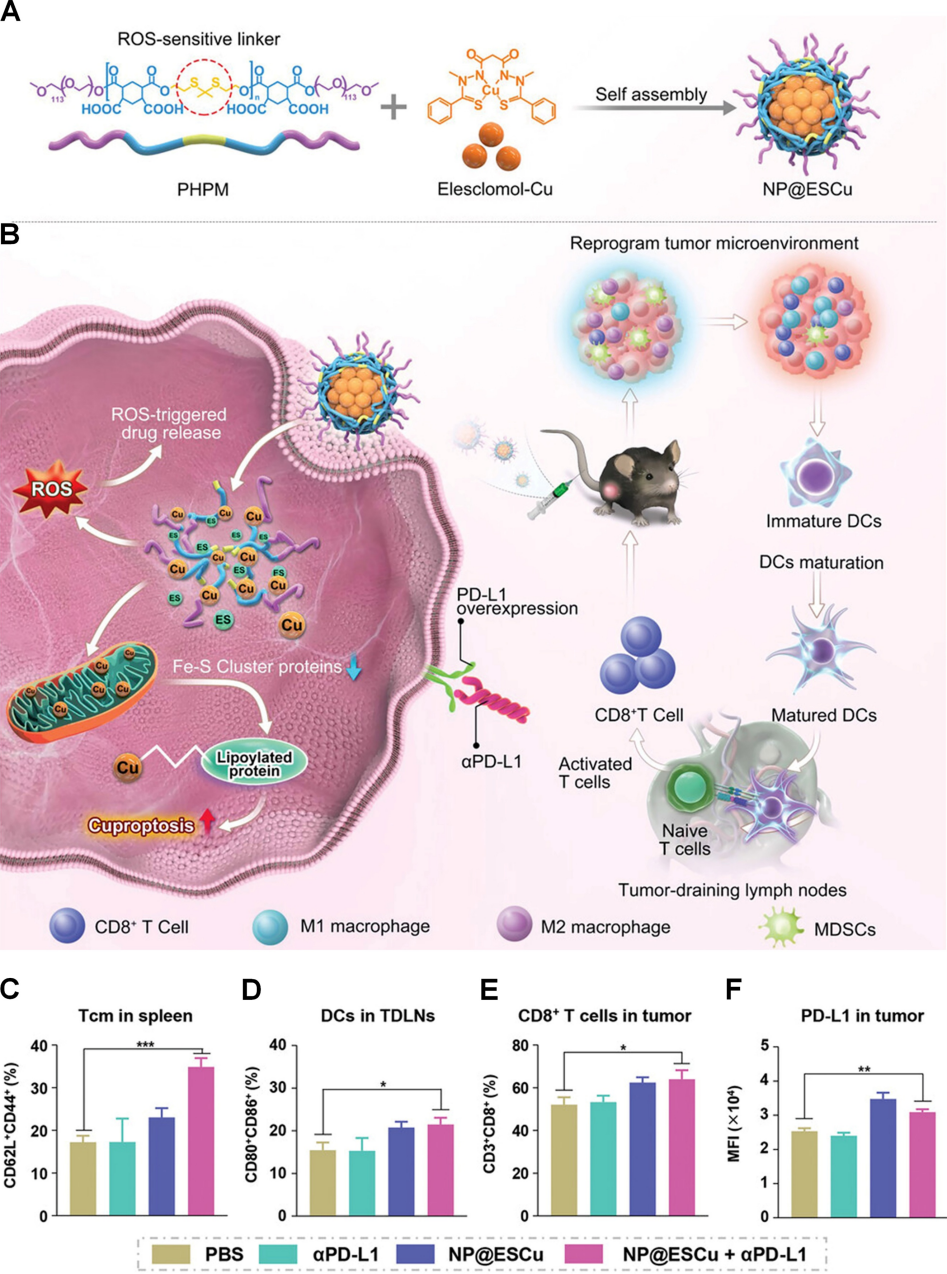
The ROS-responsive NP@ESCu potentiates the efficacy of cancer immunotherapy through the induction of cuproptosis. (A) Schematic illustration of the synthesis of NP@ESCu. (B) Schematic diagram illustrating the mechanism by which NP@ESCu elicits antitumor immune responses. (C) Proportion of central memory (CD62L^+^CD44^+^) T cells in the spleens of MB49 tumor-bearing mice across different treatment groups. (D) Proportion of mature DCs in the TDLNs of MB49 tumor-bearing mice across different treatment groups. (E) Proportion of CD8^+^ T cells in the tumors of MB49 tumor-bearing mice across different treatment groups. (F) Semi-quantitative assessment of PD-L1 expression in the tumors of MB49 tumor-bearing mice across different treatment groups. *P < 0.05, **P < 0.01, ***P < 0.001. Reproduced with permission from Wiley-VCH (journal citation: 55).

The clinical efficacy of αPD-L1 therapy in bladder cancer treatment is hindered by the immunosuppressive TME and significant interpatient variability in PD-L1 expression. Recent studies have demonstrated that cuproptosis can upregulate PD-L1 expression in tumor cells, thereby enhancing the therapeutic potential of αPD-L1-based interventions. Guo et al. synthesized a nanosystem (NP@ESCu) by loading ES and copper ions in an amphiphilic biodegradable polymer (Figure 3A) 55. In the TME, characterized by elevated levels of ROS, NP@ESCu releases ES and copper ions in a responsive manner, leading to a marked increase in intracellular copper concentrations (Figure 3B). *In vitro* experiments demonstrated that NP@ESCu effectively induced cuproptosis in BIU-87 cells by downregulating the iron-sulfur cluster protein LIAS. In a subcutaneous MB49 tumor model, NP@ESCu + αPD-L1 achieved superior antitumor efficacy compared to monotherapies, resulting in significant tumor regression. Immunological analyses conducted *via* flow cytometry indicated that this combinatorial treatment regimen substantially modulated the tumor immune microenvironment. Specifically, the percentage of central memory T cells in the spleen of mice treated with NP@ESCu combined with αPD-L1 was 34.8%, representing a nearly 2-fold increase compared to the PBS group (17.2%) (Figure 3C). The proportion of mature DCs in the TDLNs was found to exhibit a 1.4-fold increase relative to the PBS group (Figure 3D). Furthermore, combination therapy led to a 1.2-fold expansion in intratumoral CD8^+^ T cell populations compared to the PBS (Figure 3E). Moreover, the proportion of MDSCs in the tumors of mice treated with NP@ESCu + αPD-L1 was 6.9%, representing approximately a 50% reduction compared to the PBS group. Notably, PD-L1 expression in the tumors of mice receiving the combination therapy was significantly upregulated, achieving levels that were 1.2-fold higher than those observed in the PBS-treated group (Figure 3F).

Pancreatic ductal adenocarcinoma is highly resistant to αPD-L1 therapy, primarily due to its robust immune evasion mechanisms and immunosuppressive TME 113. Cuproptosis has shown promise in reversing this immunosuppressive TME by inducing ICD and modulating PD-L1 expression, thereby enhancing the efficacy of αPD-L1 therapy. Gao et al. prepared Tussah silk fibroin nanoparticles (TSF@ES-Cu NPs) through the self-assembly of ES-Cu complex, Cu^2+^, and Tussah silk fibroin 114. Notably, the incorporation of Tussah silk fibroin enables these nanoparticles to specifically target tumor cells while achieving pH-, GSH-, and ROS-responsive drug release in the TME. *In vitro* experiments demonstrated that TSF@ES-Cu NP-induced cell death could be specifically inhibited by cuproptosis inhibitors, confirming that this process is indeed mediated by cuproptosis. In a subcutaneous Pan02 mouse model, TSF@ES-Cu NPs treatment dramatically suppressed tumor growth, reducing the average tumor weight to 0.3 g, which represented only 30% of the tumor weight observed in the PBS control group. Flow cytometry analysis revealed that TSF@ES-Cu NPs + αPD-L1 obviously increased the proportion of DCs in the TME compared to either αPD-L1 or TSF@ES-Cu NPs monotherapy. Additionally, the combination treatment resulted in a 1.3-fold increase in the proportion of CD3^+^CD8^+^ T cells and a 1.2-fold reduction in M2 macrophage levels relative to TSF@ES-Cu NPs monotherapy. Notably, the spleens of mice treated with the combination treatment exhibited a 1.5-fold greater number of CD8⁺ T cells compared to those treated with TSF@ES-Cu NPs alone. This study provides a promising strategy for advancing copper-based nanomaterials as immunotherapeutic agents.

### 4.5. Cuproptosis combined with PDT

PDT represents a highly selective and minimally invasive therapeutic modality that generates ROS through the synergistic interaction of photosensitizers, light, and oxygen, ultimately leading to tumor cell destruction. The mechanism involves the photosensitizer transitioning from its ground state to an excited state upon irradiation with light of a specific wavelength. Subsequently, energy transfer occurs to neighboring oxygen molecules or substrates, producing singlet oxygen, •OH, and other ROS, which collectively induce oxidative damage to tumor cells. Due to its high specificity, minimal invasiveness, and low systemic toxicity, PDT has emerged as a promising strategy in cancer therapy. Notably, PDT-induced ICD facilitates the release of TAAs, thereby eliciting antitumor immune responses and opening novel avenues for cancer treatment 115. However, when employed as a monotherapy, the immune response triggered by PDT is generally limited, which restricts its capacity to achieve complete eradication of residual tumor cells and diminishes its therapeutic efficacy. Cuproptosis occurs upon accumulation of copper ions within tumor cells beyond their critical intracellular threshold. Tumor cells often exhibit elevated levels of H_2_O_2_, which reacts with copper ions *via* Fenton-like reaction to generate ROS. This process not only directly induces tumor cell death but also significantly enhances the efficacy of PDT. Furthermore, cuproptosis is a potent inducer of ICD, and its combination with PDT results in a synergistic enhancement of antitumor immune responses, offering improved prospects for complete tumor elimination 116. Cu^+^ plays a pivotal role as the direct triggers of cuproptosis. It is noteworthy that during PDT, superoxide anions are generated as part of the ROS cascade. These anions are capable of directly reducing Cu^2+^ to Cu^+^ without requiring additional ligands or chelators, thereby establishing the necessary conditions for cuproptosis and further augmenting tumor cell death 117. This intrinsic mechanistic link between PDT-mediated ROS generation and cuproptosis activation highlights the potential for rational design of combination therapies to maximize therapeutic outcomes in oncology.

While PDT exerts its cytotoxic effects through oxygen-dependent generation of ROS, its therapeutic efficacy is significantly constrained by the characteristic hypoxic microenvironment of solid tumors. Cuproptosis alleviates tumor hypoxia by disrupting mitochondrial metabolism and reducing oxygen consumption. Concurrently, copper ions catalyze Fenton-like reactions to generate ROS, improving oxygen availability for PDT. This dual mechanism enhances photosensitizer efficacy while overcoming hypoxia-induced treatment resistance, creating a self-amplifying therapeutic cascade against tumors 85. Liang et al. developed a copper-coordinated nanoassembly (CCNAs) through the self-assembly of Zinc Phthalocyanine-DOX prodrug (ZnPc-TK-DOX), Cu^2+^, and indoleamine 2,3-dioxygenase inhibitor 118. The CCNAs exhibited excellent stability *in vitro* and demonstrated GSH-responsive drug release behavior. Subsequent experiments confirmed their ability to effectively induce cuproptosis. In hypoxic environments, ZnPc-TK-DOX exhibited a significant reduction in ROS generation, whereas CCNAs maintained ROS levels comparable to those observed under normoxic conditions, demonstrating their robust photodynamic therapeutic efficacy even in low-oxygen TME. Furthermore, cytotoxicity assays revealed that the combination of CCNAs and laser irradiation significantly enhanced cancer cell killing compared to free ZnPc-TK-DOX. In a bilateral subcutaneous PC-3 tumor mouse model, the combination of CCNAs with laser irradiation achieved near-complete eradication of primary tumors and reduced the growth of distant tumors by 83%. Flow cytometry analysis revealed that the CCNAs + laser group exhibited a 2.1-fold increase in CD3^+^CD8^+^ T cells and a 1.5-fold increase in CD3^+^CD4^+^ T cells compared to the ZnPc-TK-DOX group. Immunofluorescence staining further confirmed a significant enhancement of CD8^+^ T cell infiltration in the CCNAs + laser group. Moreover, ELISA demonstrated that the CCNAs + laser group exhibited the highest levels of interleukin-6, TNF-α, and interferon-γ (IFN-γ) secretion. These findings collectively underscore the synergistic potential and therapeutic benefits of integrating PDT with cuproptosis in the context of cancer immunotherapy, highlighting a promising strategy for enhanced antitumor efficacy.

PDT is a promising strategy for immune activation, but the toxicity of photosensitizers limits its clinical application. Aloe-emodin (AE), a natural compound with broad-spectrum anticancer activity and excellent safety profile, demonstrates great potential as an effective photosensitizer 119. Given the limited tissue penetration of PDT, combining it with cuproptosis may synergistically enhance antitumor efficacy. Yu et al. utilized AE loaded with copper ions, which were further assembled into nanoparticles (NPs) *via* modification with PEG_2k_-DSPE-FA 116. The synthesized NPs exhibited a uniform spherical morphology with an average hydrodynamic diameter of 143.9 nm and a Zeta potential of -15 mV, as measured by DLS. The drug loading efficiencies for AE and copper ions were determined to be 58.54% and 43.32%, respectively. The NPs exhibited remarkable degradation under acidic conditions (pH 5.5), ensuring their specific drug release in tumors. *In vitro*, NPs combined with 450 nm laser irradiation significantly enhanced ROS generation and reduced intracellular GSH by 21.42% compared to AE + laser treatment. They also promoted DLAT oligomerization and downregulated FDX1 and LIAS, thus confirming the induction of cuproptosis. In an LLC tumor-bearing mouse model, NPs combined with 450 nm laser irradiation achieved a tumor inhibition rate of 55.08%. Flow cytometry revealed that compared with saline, the combined treatment increased the proportion of mature DCs in TDLNs and tumor tissues by 1.84- and 2.06-fold, respectively. Furthermore, the proportion of intratumoral CD3^+^CD8^+^ T cells in NPs + laser group was 32.73%, which was significantly higher than that in the saline group (26.5%). Moreover, the combined treatment reduced the proportion of MDSCs in tumor tissues from 23.13% in the control group to 6.43%. Additionally, CD8^+^ T cells isolated from the spleens of mice treated with NPs + laser exhibited differentiation into central memory and effector T cell subsets, indicating the establishment of durable immune memory. For each experiment, the sample size was n = 3 for *in vitro* studies and n = 5 for *in vivo* experiments. Statistical significance was determined using one-way ANOVA. Although results exhibited consistent findings across LLC cells, H1299 spheroids, and LLC mouse models, independent validation in other tumor types or laboratories remains to be reported. Collectively, these findings demonstrate that integrating PDT with cuproptosis enhances local antitumor efficacy and stimulates systemic antitumor immunity, presenting a promising strategy for cancer immunotherapy.

### 4.6. Cuproptosis combined with PTT

PTT, an innovative approach for tumor treatment, utilizes photothermal conversion materials to efficiently convert light energy into heat energy under NIR irradiation. The fundamental mechanism involves the generation of heat energy that disrupts the intracellular structure of tumor cells, leading to protein denaturation, membrane damage, and ultimately tumor cell death through ablation. This NIR light-based modality offers distinct advantages, including precise controllability, operational simplicity, and reliable biocompatibility, making it a highly promising strategy for local tumor hyperthermia. However, PTT also presents several limitations. During treatment, the upregulation of heat shock protein (HSP) diminishes therapeutic efficacy, as these proteins enhance cellular resistance to external stress, thereby reducing tumor cell sensitivity to thermal damage 120. Furthermore, single-modality PTT often fails to achieve optimal therapeutic outcomes in the complex TME. To address these limitations and enhance therapeutic efficacy, clinical research increasingly explores combination therapies integrating PTT with other treatment modalities. Recent studies have demonstrated that combining PTT with cuproptosis achieves significantly enhanced tumor-killing effects 121. Cuproptosis is characterized by mitochondrial dysfunction due to the downregulation of FDX1 and TCA cycle-related proteins, leading to elevated intracellular ROS levels. These ROS synergistically interact with those generated by PTT, exacerbating oxidative stress and amplifying tumor cell death. Furthermore, mitochondrial damage reduces ATP production, which is essential for HSP synthesis. The subsequent decrease in HSP expression renders tumor cells more vulnerable to thermal damage, thereby enhancing the efficacy of PTT 122. Notably, cuproptosis triggers the release of specific DAMPs, which activate the immune system and promote ICD. When combined with PTT-induced thermal damage, this process is further augmented, resulting in a robust systemic immune response that improves tumor recognition and elimination. This dual-modality approach not only achieves precise and potent antitumor effects but also harnesses the immune system to establish long-term cancer control, representing a promising strategy for next-generation oncology therapies.

To address copper ion leakage, nonspecific accumulation, and limited light penetration 123, Dai et al. developed a biomimetic cuproptosis inducer (PCD@CM) for synergistic photothermal immunotherapy 124. PCD@CM was synthesized through the self-assembly of NIR-II ultrasmall polymer dots, Cu^2+^, and DOX and the subsequent coating of 4T1 tumor cell membranes. The coating increased the hydrodynamic diameter from 91.3 nm (PCD nanoparticles) to 122 nm (PCD@CM). PCD@CM exhibited a high photothermal conversion efficiency of 42.36%. *In vitro* experiments revealed that PCD@CM underwent GSH-responsive disintegration. Western blot analysis demonstrated that PCD@CM treatment effectively triggered both protein lipoylation and oligomerization of DLAT. Notably, NIR laser irradiation substantially amplified the oligomerization process, suggesting that photothermally enhanced copper ion liberation potentiates cuproptosis induction. In a 4T1 tumor-bearing mouse model, PCD@CM combined with laser irradiation (PCD@CM + L) achieved the most pronounced tumor suppression. Flow cytometry analysis revealed a 3.29-fold increase in mature DCs in the PCD@CM + L group compared to the PCD@CM group alone. Notably, Treg counts in the PCD@CM+L group were reduced by 3.1 times relative to the PBS group. To further enhance therapeutic efficacy, the addition of αPD-L1 to the PCD@CM + L treatment resulted in a significant amplification of immune response, with CD8^+^ and CD4^+^ T cell populations increasing by 15.32-fold and 4.93-fold, respectively, compared to PBS group. Moreover, the number of central memory T cells in the spleen of PCD@CM + L + αPD-L1 group was found to be 4.83 times higher than that of the PBS group. Statistical analysis in this study was conducted using one-way ANOVA. Collectively, these results highlight a novel and effective strategy for enhancing copper-induced cancer cell death through NIR-II photothermal sensitization, offering new prospects for advanced cancer immunotherapy.

In TNBC, immune evasion, driven by defective ICD and a profoundly immune-suppressive TME, significantly compromises therapeutic efficacy while increasing metastatic risks and recurrence rates. To address these challenges, Jing et al. developed an innovative copper-based, self-amplifying hollow nanogenerator (termed AHPR) to potentiate ICD induction 125. Upon NIR laser irradiation, AHPR leverages a robust photothermal effect to simultaneously and effectively trigger both cuproptosis and ICD. This dual-mode mechanism enhances immune cell infiltration and activation in the TME, representing a promising therapeutic strategy for TNBC treatment.

**Figure 4:**
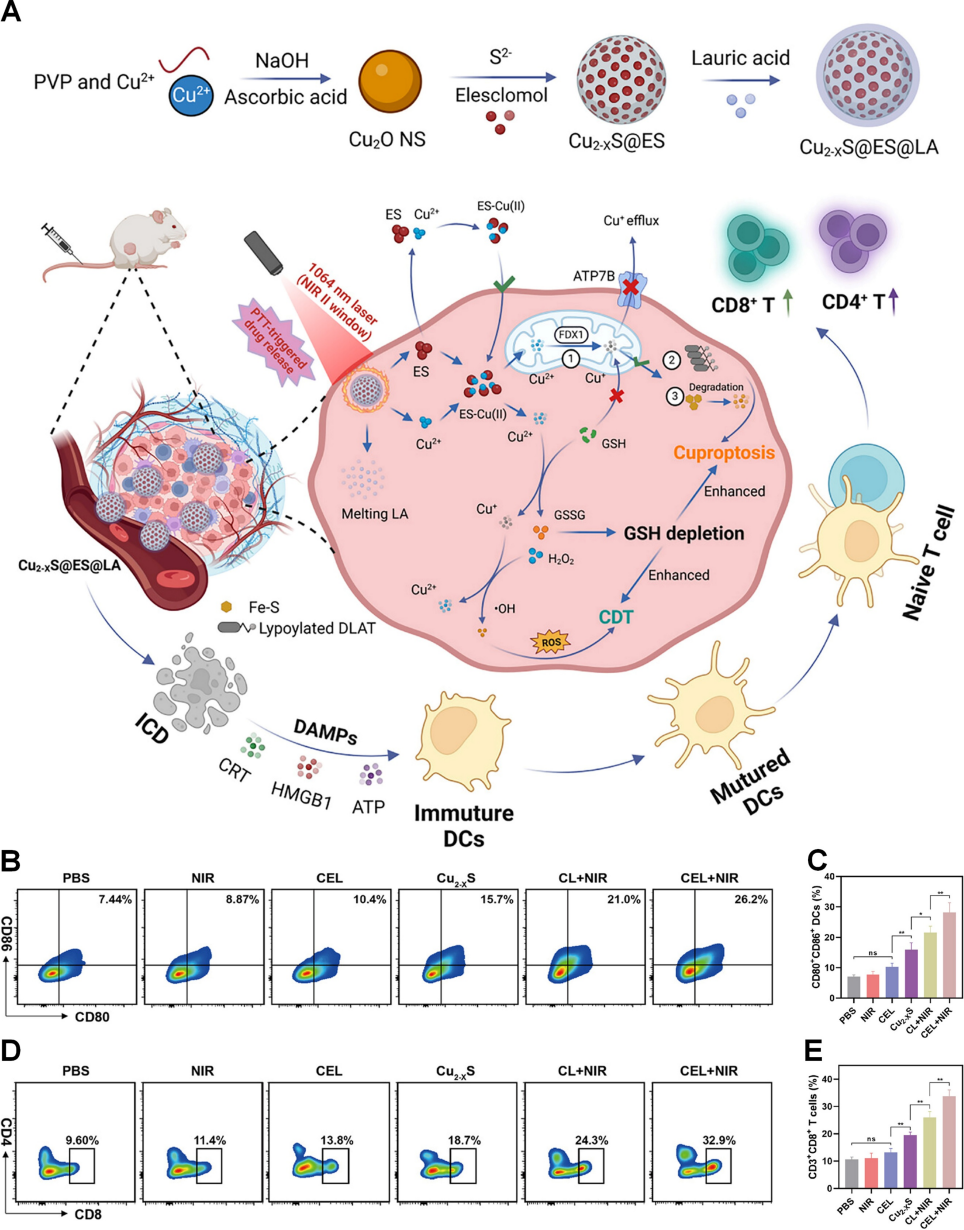
A copper-based photothermal-responsive nanoplatform induces antitumor immune responses *via* the synergistic effects of cuproptosis, PTT, and CDT. (A) Schematic illustration of the synthesis of CEL NP and their mechanism in inducing antitumor immune responses through the combined application of PTT and CDT. Representative flow cytometry images (B) and corresponding statistical analysis (C) of mature DCs in the TDLNs of CT26 tumor-bearing mice receiving different treatments. Representative flow cytometry images (D) and corresponding statistical analysis (E) of CD8^+^ T cells in the primary tumor tissues of CT26 tumor-bearing mice receiving different treatments. *P < 0.05, **P < 0.01, and ns indicates no significant difference. Reproduced with permission from Wiley-VCH (journal citation: 126).

To overcome the limitations of copper ion-mediated cuproptosis in cancer therapy, including inefficient intracellular copper delivery and off-target toxicity to healthy tissues, Cheng et al. developed a self-amplifying cuproptosis nanoplatform (CEL NP) by encapsulating ES in Cu_2_₋*_X_*S hollow nanospheres and then coating them with the phase-change material lauric acid (Figure 4A) 126. NIR irradiation triggers the disassembly of CEL NP, enabling precise release of ES and copper ions in the TME. *In vitro* experiments confirmed that the combination of laser irradiation and acidic conditions synergistically induced targeted cargo release. Subsequent studies demonstrated that CEL NP combined with NIR laser irradiation (CEL NP + NIR) significantly promoted DLAT protein aggregation and downregulated FDX1 and LIAS expression, indicating robust cuproptosis induction under photothermal stimulation. In a CT26 tumor-bearing mouse model, the CEL NP + NIR group exhibited the most pronounced tumor suppression among all treatment groups, likely attributed to the synergistic effects of cuproptosis and PTT. Infrared thermography analysis revealed that the tumor temperature in the CEL NP group reached 51.4 °C, significantly higher than that in the PBS group, demonstrating its excellent *in vivo* photothermal performance. Flow cytometry analysis of TDLNs showed that the population of matured DCs in the CEL NP + NIR group was 28.2%, which was 4.02-fold higher than that in the PBS group (Figures 4B and C). Furthermore, the infiltration of CD3^+^CD8^+^ T cells in tumors of the CEL NP + NIR group increased to 33.7%, significantly surpassing that of other subgroups (Figures 4D and E). Notably, the proportion of Tregs in the CEL NP + NIR group decreased to 9.85%, indicating a favorable shift in the immune response profile. These findings suggest that CEL NP, through targeted cuproptosis induction under photothermal conditions, represents a promising therapeutic strategy for enhancing cancer immunotherapy outcomes.

### 4.7. Cuproptosis combined with chemodynamic therapy (CDT)

CDT, an innovative and promising antitumor treatment modality, has shown remarkable potential in cancer therapy. The core principle of CDT involves the generation of highly reactive and cytotoxic ROS at tumor sites through Fenton or Fenton-like reactions. These ROS can inflict severe oxidative damage to both the structural and functional components of tumor cells, ultimately leading to tumor cell death 127. CDT has attracted increasing attention in recent years due to its numerous advantages, among which the most important ones are tumor specificity and selectivity. The TME is characterized by an acidic pH and elevated concentrations of H_2_O_2_. CDT utilizes these unique characteristics to enable the reaction to occur with greater precision at the tumor site while minimizing off-target effects. This feature not only reduces damage to healthy tissues but also significantly lowers systemic toxicity, thereby enhancing the overall therapeutic efficacy for patients 128.

For CDT, the catalytic efficiency of metal ions and the intracellular concentration of H_2_O_2_ are two critical factors influence ROS generation. While traditional iron ions are known to participate in Fenton reactions, they also exhibit certain limitations 129. In contrast, copper ions demonstrate distinct advantages in this context. Within the acidic pH range typically found in the TME, copper ions not only display higher catalytic efficiency but also operate effectively across a broader pH range for Fenton-like reactions. This unique property allows copper ions to more efficiently catalyze the production of ROS from H_2_O_2_, resulting in more severe oxidative damage to tumor cells 130. Furthermore, copper ions possess a distinctive mechanism that further enhances their therapeutic potential. On one hand, copper ions can induce cuproptosis, a novel form of cell death that is distinct from conventional apoptosis, providing new avenues for destroying tumor cells. On the other hand, Cu^2+^ released at the tumor site can be reduced to Cu⁺ by GSH, which is overexpressed in many tumors. This reduction process is particularly significant as it not only further activates cuproptosis but also depletes intracellular GSH levels 131. GSH plays a critical role in maintaining intracellular redox balance and mitigating oxidative stress. The depletion of GSH by copper ions compromises the antioxidant defense mechanisms of tumor cells, thereby amplifying the cytotoxic effects of CDT. This dual effect of promoting ROS production and disrupting cellular redox homeostasis makes copper ions particularly effective in enhancing the therapeutic effect of CDT. Notably, when cuproptosis is combined with CDT, it triggers a cascade of immune responses. This synergistic effect induces ICD and leads to the release of DAMPs. ICD initiates a specific antitumor immune response, activating the host's immune system to recognize and eliminate cancer cells more effectively. This immunotherapeutic feature is expected to play a pivotal role in augmenting antitumor immunotherapy, offering new and exciting possibilities for advancing cancer treatment strategies.

Copper-based CDT agents have demonstrated remarkable antitumor efficacy due to their robust Fenton-like catalytic activity. The integration of CDT with cuproptosis in copper-based nanomedicines represents a promising strategy to enhance cancer treatment outcomes. Xu et al. encapsulated lactate oxidase (LOx) in yolk-like CuO@Gd_2_O_3_ nanoparticles to prepare CGYL-LOx, and further cloaked them with Renca cell membranes to form biomimetic mCGYL-LOx particles 132. The size of mCGYL-LOx observed by transmission electron microscope was 190 nm and the loading efficiency of LOx was 77.3%. In the TME, LOx catalyzes the oxidation of excess lactate into H_2_O_2_, which is further converted into cytotoxic •OH, thereby executing CDT. Concurrently, the release of copper ions not only induces cuproptosis but also amplifies ROS generation through Fenton-like reactions. *In vitro* experiments revealed that mCGYL-LOx nanoparticles induced significant copper overload and ROS bursts, leading to substantial mitochondrial damage and initiation of cuproptosis. Confocal microscopy and flow cytometry analyses confirmed the efficient clathrin-mediated endocytosis of these nanoparticles by Renca cells. In a Renca tumor-bearing mouse model, the mCGYL-LOx treatment group exhibited the most pronounced tumor suppression. Flow cytometry analysis demonstrated increased infiltration of CD4^+^ T cells, CD8^+^ T cells, and NK cells in the tumor tissues of the mCGYL-LOx treatment group, accompanied by a significant reduction in Tregs. Notably, the proportion of mature DCs in the spleen of the mCGYL-LOx treatment group was approximately 4.8-fold that of the saline treatment group. Overall, this study presents a novel and effective approach for renal cancer immunotherapy by synergistically integrating CDT with cuproptosis in a copper-based nanoplatform.

**Figure 5:**
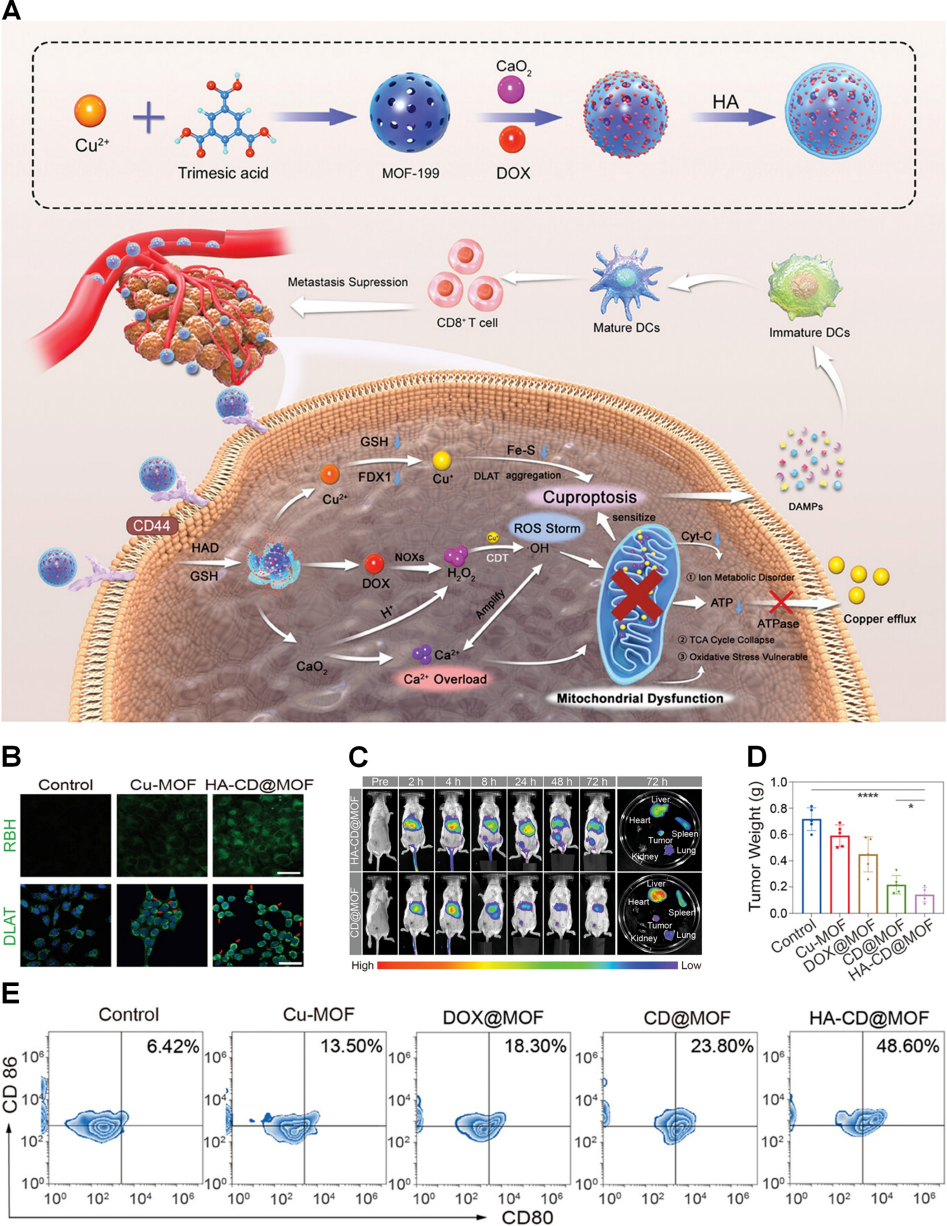
A bimetallic mitochondrial disruptor promotes antitumor immunotherapy through the induction of cuproptosis in combination with CDT. (A) Schematic illustration of the preparation of HA-CD@MOF and their mechanism in activating antitumor immune responses. (B) Confocal laser scanning microscopy images depicting intracellular Cu^2+^ accumulation and DLAT aggregation in 4T1 cells following different treatments. (C) *In vivo* fluorescence images of 4T1 tumor-bearing mice captured at various time points after different treatments and *ex vivo* fluorescence images of major organs and tumors collected 72 h post-treatment. (D) Average tumor weights of 4T1 tumor-bearing mice measured on day 15 across different treatment groups. (E) *In vivo* assessment of DC maturation in 4T1 tumor-bearing mice across different treatment groups. *P < 0.05, ****P < 0.0001. Reproduced with permission from Wiley-VCH (journal citation: 133).

Exogenous copper has been shown to synergistically enhance CDT and induce cuproptosis. However, elevated expression of copper transporters in tumor cells promotes copper efflux, thereby limiting therapeutic efficacy. Previous studies have demonstrated that excessive intracellular accumulation of Ca^2+^ can induce mitochondrial dysfunction and disrupt the energy-dependent copper efflux, thereby increasing intracellular copper levels. Du et al. developed a self-amplifying bimetallic mitochondrial disruptor (HA-CD@MOF) through a one-pot synthesis approach (Figure 5A) 133. Specifically, HA-CD@MOF was synthesized by encapsulating DOX and calcium peroxide in HA-modified copper-based MOF. In the TME with high levels of GSH and hyaluronidase, HA-CD@MOF disintegrates and releases payloads. Calcium peroxide decomposes under mildly acidic conditions, releasing H_2_O_2_ and Ca^2+^. The released H_2_O_2_ enhances Cu^2+^-mediated CDT, while Ca^2+^ inhibits Cu-ATPase activity, promoting intracellular copper accumulation and triggering cuproptosis. *In vitro* experiments demonstrated that HA-CD@MOF significantly reduced Cu-ATPase activity and markedly increased intracellular Cu^2+^ levels, effectively inducing cuproptosis (Figure 5B). According to the fluorescence imaging results, HA effectively increased the intratumoral accumulation of HA-CD@MOF by prolonging its residence time (Figure 5C). *In vivo* studies using 4T1 tumor-bearing mice showed that HA-CD@MOF achieved the highest tumor growth inhibition rate (72.45%) and reduced tumor weight to approximately one-fifth of that in the control group (Figure 5D). Flow cytometry analysis revealed a significant increase in the proportion of mature DCs in tumor tissues of the HA-CD@MOF treatment group, which was approximately 7.6-fold higher than that of the control group (Figure 5E). Subsequent ELISA assays demonstrated elevated levels of interleukin-6, TNF-α, and IFN-γ in the HA-CD@MOF treatment group. Furthermore, in a lung metastasis model, the HA-CD@MOF treatment group exhibited the fewest pulmonary nodules. These findings highlight the therapeutic potential of HA-CD@MOF as a nanoplatform that integrates CDT and cuproptosis to effectively target both primary and metastatic tumors.

The high expression of indoleamine 2,3-dioxygenase 1 (IDO1) in tumors facilitates the conversion of tryptophan to kynurenine, thereby promoting the establishment of a tumor immune-suppressive microenvironment. This process impairs immune cell function, enabling tumor immune evasion and limiting the efficacy of immunotherapy. As a highly selective IDO1 inhibitor, NLG919 has demonstrated potential in reversing tumor immune evasion 134. Wu et al. developed a biomimetic nanomaterial (ECNM) that integrates ES, Cu^2+^, and NLG919 through self-assembly, followed by surface coating with 4T1 cell membranes 135. This biomimetic design allows ECNM to achieve active tumor targeting through homotypic adhesion and immune evasion, thereby enhancing tumor accumulation. *In vitro* experiments revealed that ECNM, upon tumor internalization, releases ES, Cu^2+^, and NLG919 in response to intracellular GSH stimulation, thus inducing cuproptosis and initiating CDT through the Fenton-like reaction of Cu^2+^. Western blot analysis demonstrated that ECNM significantly downregulated IDO1 expression in 4T1 tumor cells, indicating its potential to reverse the tumor immune microenvironment. In a 4T1 tumor-bearing mouse model, ECNM showed the most pronounced antitumor effects. 66.7% of mice in the ECNM treatment group survived for up to 40 days. Flow cytometry analysis revealed that ECNM increased the proportion of mature DCs in TDLNs by approximately 3.96-fold and 1.46-fold compared to the ES + Cu^2+^ groups and ES + Cu^2+^ + NLG919 groups, respectively. Furthermore, ECNM effectively inhibited the infiltration of Tregs into tumors. In a bilateral tumor model, flow cytometry demonstrated that ECNM promoted DCs maturation in TDLNs and enhanced CD8^+^ T cell infiltration. Collectively, these findings highlight ECNM as an effective strategy for cancer immunotherapy.

### 4.8. Cuproptosis combined with sonodynamic therapy (SDT)

SDT, a non-invasive therapeutic modality, employs ultrasound to activate sonosensitizers, generating ROS that induce oxidative stress and trigger tumor cell death. SDT offers distinct advantages, including non-invasiveness, deep tissue penetration, and minimal systemic toxicity. Compared with conventional radiotherapy and chemotherapy, SDT provides more precise tumor targeting while minimizing collateral damage to surrounding normal tissues and demonstrates promising efficacy in the ablation of deep-seated tumors. Nevertheless, the efficacy of SDT is frequently limited by elevated GSH levels in tumor cells, which rapidly scavenge ROS and diminish oxidative stress, thereby attenuating its antitumor effects 136. Cuproptosis provides a novel strategy to overcome this limitation. On one hand, excess copper ions can deplete aberrantly elevated GSH in tumor cells, weakening the antioxidant defense system and amplifying SDT-induced oxidative stress 16. On the other hand, SDT-mediated ROS generation exacerbates protein misfolding and mitochondrial stress, reinforcing the cytotoxic mechanisms underlying cuproptosis 137. This mutual reinforcement between cuproptosis and SDT not only enhances direct tumoricidal activity but also has significant immunological implications. Specifically, the combined approach induces robust ICD, which promotes DC maturation and primes cytotoxic T cell responses. This systemic antitumor immune activation helps overcome resistance to single-modality SDT, offering a promising avenue for durable cancer immunotherapy. Therefore, the integration of cuproptosis and SDT establishes a synergistic therapeutic paradigm. Copper-mediated GSH depletion sensitizes tumors to ROS generated by SDT, while SDT-induced oxidative and proteotoxic stress strengthens cuproptosis pathways, ultimately leading to augmented tumor suppression and immune activation.

**Figure 6:**
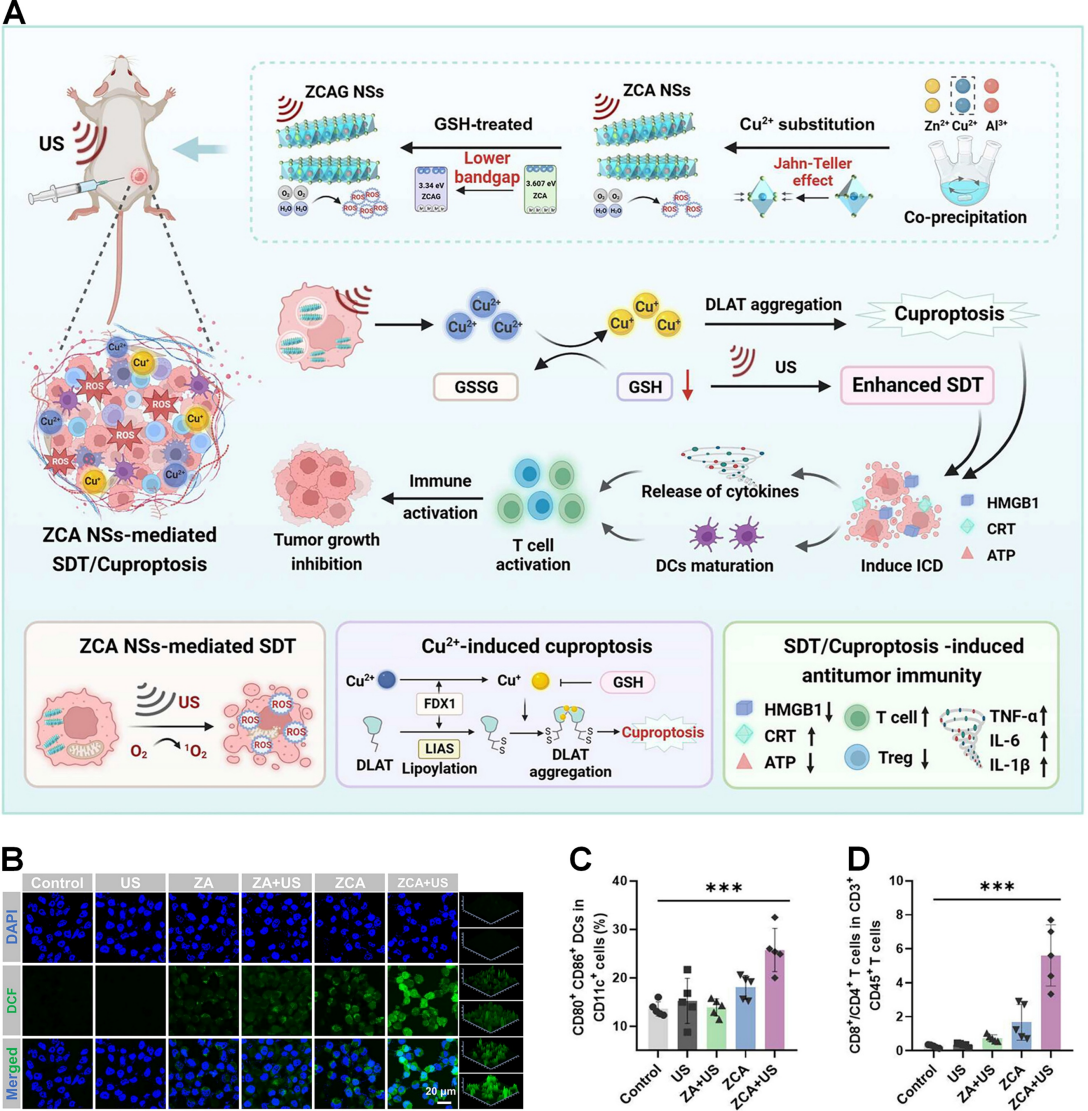
Bioactive layered double hydroxides potentiate antitumor immunotherapy *via* the synergistic effects of SDT and cuproptosis. (A) Schematic illustration of the potential mechanism by which ZCA NSs mediate the synergistic effects of SDT and cuproptosis in inducing antitumor immune responses. (B) Confocal images of CT26 cells stained with DCFH-DA, demonstrating intracellular ROS generation following various treatments (ZA NSs/ZCA NSs: 50 ppm; ultrasound irradiation: 30 kHz, 3 W/cm^2^, 2 min). (C) Flow cytometric quantification of DC maturation in lymph nodes of CT26 tumor-bearing mice receiving different treatments. (D) Flow cytometric quantification of CD8^+^/CD4^+^ T cells in the tumors of CT26 tumor-bearing mice receiving different treatments. ***P < 0.001. Reproduced with permission from American Chemical Society (journal citation: 139).

Conventional sonosensitizers are limited by several challenges, including poor stability, phototoxic side effects, and reduced therapeutic efficacy due to the presence of intracellular antioxidants such as GSH 138. Additionally, the insufficient copper concentration in tumor cells often hinders the effective induction of cuproptosis. To address these limitations, the development of multifunctional nanoplatforms capable of simultaneously initiating SDT and delivering copper ions represents a highly promising strategy. Tang et al. synthesized copper-substituted zinc-aluminum layered double hydroxide nanosheets (ZCA NSs) using a co-precipitation method (Figure 6A) 139. Under ultrasound exposure, ZCA NSs effectively generated abundant ROS and depleted intracellular GSH, thereby intensifying oxidative stress and achieving potent SDT efficacy (Figure 6B). *In vitro* experiments demonstrated that ZCA NSs successfully induced cuproptosis, with the effect significantly enhanced under ultrasound exposure. Furthermore, imaging studies using Cy5.5-labeled ZCA NSs confirmed their efficient cellular internalization *via* endocytosis. *In vivo* evaluations were conducted in a CT26 colon cancer mouse model, where the combination of ZCA NSs and ultrasound resulted in complete tumor eradication within four days, with no tumor recurrence or mortality observed over two months. Flow cytometry analysis revealed that the proportion of mature DCs in lymph nodes was 25.8% in the ZCA NSs combined with ultrasound group, significantly higher than the 13.6% observed in the control group (Figure 6C). Additionally, the ratio of CD8⁺ CTLs to CD4⁺ helper T lymphocytes in the tumor were substantially elevated in the ZCA NSs + ultrasound group, indicating robust activation of antitumor immune responses (Figure 6D). To further validate the therapeutic potential of this approach, experiments were conducted in a low-immunogenic, highly metastatic 4T1-luc breast cancer mouse model. H&E staining and bioluminescence imaging revealed extensive lung and liver metastases in both the control and ultrasound monotherapy groups, whereas no metastatic lesions were detected in the liver or lungs of mice treated with ZCA NSs + ultrasound.

The Warburg effect drives tumor cells to preferentially metabolize glucose into lactate through glycolysis even in oxygen-rich conditions. This excessive lactate accumulation not only promotes tumor progression by activating signaling pathways that enhance invasion, metastasis, and angiogenesis but also confers resistance to RT and chemotherapy, suppresses cytotoxic T cell activity, and facilitates immune evasion 140. LOx, an enzyme that catalyzes the oxidation of lactate to H_2_O_2_, has emerged as a promising therapeutic agent for cancer therapy. Yu et al. developed a semiconductor polymer-based nanoreactor (SPN_LCu_) for targeted pancreatic cancer therapy 141. Specifically, LOx was anchored to sonodynamic polymer nanoparticle (SPN) *via* a ROS-cleavable linker, and the resulting complex was further coordinated with Cu^2+^ ions to form SPN_LCu_. For comparison, they also prepared LOx-conjugated SPNs (SPN_L_) and Cu^2+^-chelated SPNs (SPN_Cu_). Upon ultrasound irradiation, the semiconducting polymer generated singlet oxygen, which cleaved ROS-responsive linkers, thereby initiating LOx release. The released LOx consumes lactate to yield H_2_O_2_ and Cu^2+^ is reduced to Cu^+^, driving •OH production and establishing a positive-feedback cascade that amplifies both LOx release and oxidative stress. This dual mechanism induces cuproptosis and elicits the hallmarks of ICD. *In vitro* experiments demonstrated that the SPN_LCu_ group achieved a cumulative LOx release rate of 91.4% and a lactate degradation efficiency of 80.7%, both significantly higher than those observed in control groups. *In vivo*, treatment with SPN_LCu_ and ultrasound suppressed subcutaneous Panc02 tumors with an inhibition rate of 80.3%, and all mice in this group survived until day 40. Flow cytometry revealed that DC maturation was modestly enhanced in both SPN_Cu_ and SPN_LCu_ groups without ultrasound treatment. However, a more pronounced effect was observed upon ultrasound application, with the SPN_LCu_ plus ultrasound group achieving the highest DC maturation rate of 35.2%. Similarly, the infiltration of CD3^+^CD4^+^ helper T cells and CD3^+^CD8^+^ cytotoxic T cells into tumors was significantly augmented following ultrasound-triggered treatments. Among these groups, SPN_LCu_ combined with ultrasound elicited the most robust immune response, with CD8^+^ T-cell tumor infiltration increasing to 26.8%, compared to 21.2% for SPN_Cu_ plus ultrasound and 17.9% for SPN_L_ plus ultrasound. These findings demonstrate that SPN_LCu_ not only mediated tumor regression but also reshaped the immune microenvironment.

B-cell lymphoma, a highly heterogeneous and aggressive hematological malignancy, frequently exhibits resistance to conventional therapies, leading to poor clinical outcomes. Given that epigenetic dysregulation plays a pivotal role in the pathogenesis of B-cell lymphoma, therapeutic strategies targeting epigenetic pathways have attracted significant attention as promising interventions 142. To this end, Wang et al. developed a novel MOF-based nanosonosensitizer, CRUPPA19, for ultrasound-triggered cascade immunotherapy of B-cell lymphoma 143. A copper-rhein complex was encapsulated in UiO-66-NH_2_ to form CuR@UiO66, which was subsequently functionalized with mPEG-PO_3_ and anti-CD19 antibodies to yield CRUPPA19 nanoparticles. *In vitro* experiments demonstrated that CRUPPA19 could selectively target autophagosomes, enabling efficient intracellular release of therapeutic agents. Immunohistochemical staining revealed elevated levels of lipoylated proteins in tumor cells following CRUPPA19 + ultrasound treatment, confirming the successful induction of cuproptosis. In a bilateral A20 tumor model, the CRUPPA19 + ultrasound group achieved near-complete regression of primary tumors and exhibited a remarkable 90.4% inhibition of distant tumor growth. Flow cytometry analysis revealed that the proportion of mature DCs in the combined treatment group increased to 54.3%, representing the highest level among all experimental groups. This was accompanied by a significant infiltration of CD8^+^ T cells and peak serum levels of IFN-γ, indicating a robust immune response. Notably, CRUPPA19 + ultrasound treatment led to marked downregulation of PD-L1 expression in tumor tissues, thereby enhancing T cell activation. These findings collectively demonstrate that CRUPPA19-mediated sonodynamic immunotherapy offers a novel and highly promising approach for the treatment of B-cell lymphoma. In another study, Cao et al. developed a Z-scheme heterojunction structure (CMC) by *in situ* growth of a copper-based MOF protective layer on Cu_2_O nanocubes 144. The results demonstrated that CMC could selectively release copper ions in tumor cells to induce cuproptosis and simultaneously elicited a synergistic antitumor response through the integration of multiple therapeutic modalities, including cuproptosis, SDT, CDT, and immunotherapy.

### 4.9. Cuproptosis combined with pyroptosis

Pyroptosis, a distinct mode of programmed cell death mediated by the gasdermin (GSDM) protein family, is characterized by membrane pore formation, leading to osmotic disequilibrium and subsequent cellular swelling. This process induces ICD through the rapid release of DAMPs, which significantly enhances tumor immunogenicity and activates a systemic immune response 145. Recent studies have revealed that cuproptosis shares a closely related molecular cascade with pyroptosis. During the process of cuproptosis, excessive intracellular copper ions catalyze Fenton-like reactions, generating substantial amounts of ROS. These ROS activate the NLRP3 inflammasome/caspase-1 signaling axis, ultimately resulting in cleavage of the GSDMD protein and activation of the pyroptotic pathway 146. Cellular copper homeostasis is primarily maintained by the copper transport system, which includes copper transporters and copper-transporting ATPases. Among these, ATP7A/B homologous proteins play a critical physiological role in mediating cellular copper efflux through ATP hydrolysis-driven transmembrane transport, thereby preventing the toxic effects associated with intracellular copper overload. Notably, pyroptosis and cuproptosis exhibit a bidirectional regulatory relationship. Specifically, the pyroptotic process impairs cellular copper efflux by inhibiting Cu-ATPase activity, leading to increased intracellular copper ion concentrations and forming a positive feedback loop that amplifies the cuproptotic effect 147. Simultaneously, both cell death pathways can independently induce ICD. Their synergistic action effectively reshapes the TME, transforming immunosuppressive "cold tumors" into "hot tumors" characterized by robust immune cell infiltration 76. This process also activates a systemic immune response capable of establishing long-lasting antitumor memory. This therapeutic strategy, based on the synergistic effect of programmed cell death pathways, overcomes the limitations of conventional therapies by reprogramming the immune microenvironment through multi-target regulation. Consequently, it provides novel theoretical foundations and transformative directions for advancing tumor immunotherapy.

Although copper-based nanoparticles can induce oxidative stress and cuproptosis in cancer cells, their therapeutic efficacy is often limited by the dynamic intracellular redox environment and the metabolic adaptability between oxidative phosphorylation and glycolysis. To overcome these challenges, Cun et al. developed a novel copper-α-ketoglutarate nanocoordination complex for colorectal cancer therapy 148. This complex was further engineered with polydopamine and PEG to enhance its physiological stability and tumor specificity. The resulting nanoplatform, termed CKPP, is designed to achieve synergistic pyroptosis-cuproptosis immunotherapy. *In vitro* experiments demonstrated that CKPP treatment led to increased expression of cleaved caspase-8 and GSDMC N-terminal fragments in CT26 cells, suggesting the induction of pyroptosis. Concurrently, the aggregation of DLAT confirmed the activation of cuproptosis pathways. Additionally, CKPP treatment significantly depleted intracellular GSH levels, increased intracellular ROS, and impaired mitochondrial ATP synthesis. *In vivo* studies utilizing a subcutaneous colorectal cancer model revealed that CKPP exhibited robust tumor-targeting capabilities with preferential accumulation in tumor tissues. Furthermore, CKPP combined with light irradiation (CKPP + L) achieved an impressive tumor inhibition rate of 96.3%, almost achieving complete tumor eradication. Notably, CKPP + L treatment triggered the release of pro-inflammatory cytokines interleukin-1β and interleukin-18, as well as HMGB1, indicating potent activation of antitumor immune responses. Flow cytometry analysis provided further evidence of immune activation, showing a marked increase in mature DCs in the spleen and a significant infiltration of CD3^+^CD4^+^ and CD3^+^CD8^+^ T lymphocytes in tumor tissues. Collectively, these findings highlight the remarkable antitumor efficacy of CKPP, which operates through the dual induction of cuproptosis and pyroptosis.

Tumor recurrence is predominantly driven by the persistence of dormant cancer cells, which evade immune detection due to their quiescent state and low immunogenicity. Harnessing robust immune responses represents a promising therapeutic strategy for eliminating residual cancer cells and preventing tumor relapse. Qiao et al. engineered a copper-quinone-GOx nanoplatform (CQG NPs) by integrating a copper-quinone cascade nanozyme with GOx 91. CQG NPs exhibit multiple enzyme-like properties, including intracellular GSH depletion, oxygen generation, and ROS production, thereby synergistically inducing cuproptosis and pyroptosis. The hydrodynamic diameter and Zeta potential of CQG NPs detected by DLS were 255 nm and +19.8 mV, respectively. In a GSH-rich medium, the release of GOx from CQG NPs reached 89.5% after 6 h. In 4T1 tumor-bearing mice, CQG NPs selectively accumulated in tumor tissues, achieving a remarkable 91.10% inhibition rate and near-complete suppression of tumor growth. CQG NPs treatment downregulated FDX1 and LIAS, promoted DLAT aggregation, upregulated NLRP3, and activated caspase-1 and cleaved GSDMD N-terminal fragments, demonstrating effective induction of both cuproptosis and pyroptosis *in vivo*. Immunological evaluations revealed that CQG NPs treatment significantly reprogrammed the TME. Specifically, the proportion of immunosuppressive M2 macrophages decreased from 25% (control group) to 12.40%, while pro-inflammatory M1 macrophages increased from 9.60% (control group) to 21.20%. CQG NPs upregulated the percentage of mature DCs in the TDLNs from 16.50% (control group) to 38.30%. Furthermore, CD4^+^ and CD8^+^ T cell populations in the spleen of the CQG NPs treatment group were significantly higher than those of the control group, accompanied by a substantial increase in both effector memory and central memory T cell subsets. These results highlight the potential of CQG NPs to reprogram the immunosuppressive TME and trigger potent immune responses capable of eliminating dormant cancer cells and preventing recurrence.

The combination of cuproptosis and pyroptosis has emerged as a promising strategy to enhance antitumor immunotherapy. However, off-target toxicity remains a critical challenge due to potential damage to healthy tissues. To address this limitation, Xiao et al. designed Pluronic F127-modified MOF-199 nanoparticles (F^127^MOF-199 NPs), a dual-responsive nanoplatform that is selectively activated by GSH and hydrogen sulfide in the TME 149. *In vitro* experiments demonstrated that F^127^MOF-199 NPs effectively induced both cuproptosis and pyroptosis in tumor cells. Fluorescence imaging with Cy5.5 revealed that F^127^MOF-199 NPs underwent GSH-responsive disassembly, confirming their tumor-specific activation. Furthermore, *in vivo* studies using CT26 tumor-bearing mice showed that F^127^MOF-199 NPs combined with laser irradiation (F^127^MOF-199 + L) substantially reduced tumor volume and tumor weight, highlighting their potent antitumor efficacy. Flow cytometry analysis demonstrated a notable enhancement in antitumor immune responses. Specifically, the proportion of mature DCs in TDLNs was 38.2% in the F^127^MOF-199 + L group, representing a 1.7-fold elevation compared to the control group. Similarly, the proportion of activated CD8^+^ T cells in the spleen exhibited a 1.6-fold increase following treatment with F^127^MOF-199 + L relative to the control group. These findings collectively underscore the capacity of F^127^MOF-199 NPs to enhance antitumor immune responses through the simultaneous induction of cuproptosis and pyroptosis, offering a promising therapeutic strategy for cancer immunotherapy.

**Figure 7:**
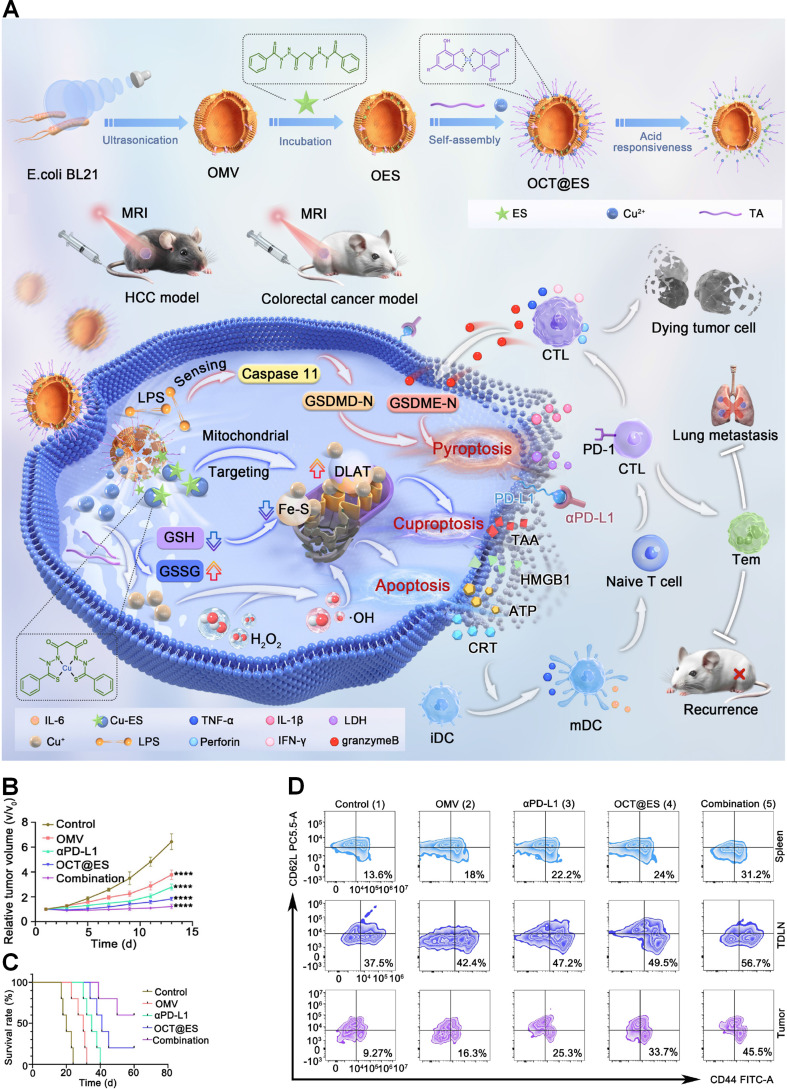
OCT@ES elicits antitumor immune responses by synergistically inducing cuproptosis, pyroptosis, and apoptosis. (A) A schematic illustration depicts the synthesis of OCT@ES and the proposed mechanism through which the coordinated induction of cuproptosis, pyroptosis, and apoptosis collectively enhances the antitumor immune response. (B) The relative tumor growth curve of the CT26 mouse model in different treatment groups. Data are shown as the mean values ± SD (n = 5) (C) The long-term survival rates of CT26 tumor-bearing mice receiving various treatments. (D) Representative flow cytometry analysis illustrates the distribution of memory effector T cells in the spleen, TDLNs, and intratumoral regions following different therapeutic interventions. ****P < 0.0001. Reproduced with permission from Ivyspring International Publisher (journal citation: 150).

Inducing multiple forms of cell death to eliminate tumor cells while simultaneously activating antitumor immunity represents a highly promising therapeutic strategy. In a recent study, Luo et al. developed a novel multimodal nanoplatform, termed OCT@ES, which integrates cuproptosis, pyroptosis, and apoptosis to achieve comprehensive tumor cell eradication (Figure 7A) 150. The hydrophobic drug ES was encapsulated in outer membrane vesicles derived from *Escherichia coli*. Subsequently, a Cu-tannic acid complex, formed through coordination between Cu^2+^ ions and tannic acid, was deposited onto the surface of outer membrane vesicles, yielding the final OCT@ES nanodelivery system. *In vitro* experiments demonstrated that OCT@ES exhibited a pH-responsive release profile, enabling precise intracellular delivery of both Cu^2+^ and ES to tumor cells. Mechanistic investigations revealed that treatment with OCT@ES upregulated the expression of caspase-11 and GSDMD-N, induced DLAT aggregation, and downregulated LIAS and FDX1 expression, thereby confirming the induction of cuproptosis and pyroptosis. In CT26 tumor-bearing mice, administration of OCT@ES resulted in significant inhibition of tumor growth (Figures 7B and C). Flow cytometry analysis demonstrated that OCT@ES treatment markedly increased the infiltration of intratumoral CD8^+^ T cells, which was significantly higher than that in the control group. Furthermore, compare to the control group, the number of mature DCs in the OCT@ES-treated group increased by 2.16-fold in the spleen, 1.57-fold in the TDLNs, and 2.45-fold in the tumor tissues. When combined with αPD-L1 treatment, the number of mature DCs in all examined tissues showed a further increase, likely due to the synergistic immune-activating effects of OCT@ES and αPD-L1. Notably, in the OCT@ES + αPD-L1 group, the proportion of Tregs in the TDLNs was reduced by nearly 50% compared to the control group. These findings highlight the robust therapeutic efficacy of the combined OCT@ES and αPD-L1 strategy in enhancing antitumor immune responses. In the tumor-rechallenge model, flow cytometry analysis showed that compared with the control group, the proportion of effector memory T cells in the spleen and TDLNs was moderately increased following treatment with OMV, αPD-L1 or OCT@ES as a single therapy. Notably, this increase was substantially enhanced in the combination therapy group, demonstrating a significant 2.3-fold elevation in the spleen and a 1.5-fold increase in the TDLNs (Figure 7D).

The overexpression of polyamines in tumor cells has been implicated in promoting tumor progression through multiple mechanisms, suggesting that reducing intracellular polyamine levels represents a promising antitumor strategy 151. Piceatannol, a natural compound with potent biological activity, inhibits polyamine synthesis by targeting arginase 2 152. Zhu et al. developed multifunctional nanoparticles (Cu-Pic/HA NPs) composed of copper, piceatannol, and HA to simultaneously deplete polyamines and enhance antitumor immune responses mediated by cuproptosis and pyroptosis 153. Cu-Pic/HA NPs achieve tumor selectivity *via* HA-mediated CD44 receptor recognition and TME-responsive biodegradation, thereby ensuring preferential accumulation and on-site activation in tumor tissues. *In vitro* studies revealed that these nanoparticles disrupt the polyamine transporter ATP13A2 on lysosomes, thereby impairing the efficient transport of polyamines within cells. Further studies revealed that Cu-Pic/HA NPs downregulate the expression of copper efflux proteins, leading to the accumulation of intracellular copper ions and subsequent induction of cuproptosis. Additionally, these nanoparticles were shown to induce pyroptosis *via* classical pathways by depleting polyamine levels. The antitumor efficacy of Cu-Pic/HA NPs was evaluated in a 4T1 tumor-bearing mouse model, where they exhibited significant therapeutic effects. Compared to the control group, the maturation rate of DCs in the Cu-Pic/HA NPs group increased 2.2-fold. Notably, treatment with Cu-Pic/HA NPs resulted in a 4.2-fold and 3.2-fold increase in the proportions of CD4^+^ and CD8^+^ T cells in the tumor, respectively, compared to the control group. Importantly, a marked reduction in Treg populations was also observed in the Cu-Pic/HA NPs-treated group. These findings collectively highlight the robust antitumor potential of Cu-Pic/HA NPs and their capacity to modulate immune responses in the TME.

### 4.10. Cuproptosis combined with ferroptosis

Ferroptosis represents a unique iron-dependent non-apoptotic form of programmed cell death, characterized by the pathological accumulation of lipid peroxides (LPO) and the dysfunction of the GSH peroxidase 4 (GPX4) antioxidant system 154. The underlying mechanism primarily involves the Fenton reaction, which is triggered by iron overload, leading to the excessive generation of reactive ROS. These ROS subsequently oxidize polyunsaturated fatty acids, resulting in the formation of LPO and ultimately compromising cellular membrane integrity. Notably, ferroptosis offers a significant advantage over traditional apoptosis in that it can bypass resistance mechanisms employed by cancer cells with defective apoptotic signaling pathways. Moreover, as a form of ICD, ferroptosis facilitates the release of tumor antigens and promotes DC maturation, thereby activating antitumor immune responses 155. However, tumor cells have evolved adaptive strategies to counteract ferroptosis. Tumor cells often exhibit elevated expression of GPX4 or compensatory upregulation of GSH. These factors contribute to the attenuation of ferroptosis: GPX4 directly neutralizes LPO, while GSH serves as an essential coenzyme for GPX4-mediated antioxidant activity. These adaptive responses can significantly impair the therapeutic efficacy of ferroptosis-based interventions 156. Recent studies have demonstrated that combining ferroptosis with cuproptosis offers a promising strategy to overcome these limitations. Cuproptosis not only depletes intracellular GSH but also disrupts cellular redox homeostasis, thereby abolishing GPX4-mediated suppression of ferroptosis 157. Importantly, the reaction between Cu^2+^ and GSH generates Cu^+^, which enhances the catalytic efficiency of Fenton-like reactions, intensifying lipid peroxidation and amplifying oxidative stress-induced damage of ferroptosis. Compared to cuproptosis alone, the concurrent induction of both cuproptosis and ferroptosis exerts significantly enhanced tumoricidal effects through convergent stress mechanisms. Specifically, acetylated protein aggregation and mitochondrial dysfunction triggered by cuproptosis synergize with ferroptosis-mediated membrane lipid peroxidation, collectively leading to irreversible multi-organelle failure 158. This combinatorial approach not only enhances direct cytotoxicity but also amplifies ICD, resulting in more robust DC maturation and greater infiltration of CD8^+^ CTLs compared to either pathway alone. While cuproptosis alone effectively disrupts mitochondrial metabolism and redox homeostasis, its combination with ferroptosis broadens the spectrum of cellular vulnerabilities and overcomes tumor metabolic plasticity, thereby providing a more potent and durable therapeutic strategy.

Therapeutic strategies that integrate multiple cell death mechanisms represent a promising strategy in cancer treatment. Ji et al. developed a multifunctional copper-based MOF nanoparticle (M/A@MOF@CM), which was cloaked with tumor cell membranes and co-loaded with mitoxantrone and axitinib 29. By leveraging homologous adhesion and immune evasion conferred by tumor cell membrane camouflage, M/A@MOF@CM achieves selective tumor targeting. *In vitro* experiments demonstrated that M/A@MOF@CM combined with laser irradiation (M/A@MOF@CM + L) resulted in significant reductions in FDX1 and LIAS expression levels, accompanied by elevated intracellular ROS and LPO, indicative of both cuproptosis and ferroptosis. *In vivo* distribution assay confirmed that M/A@MOF@CM has high tumor-targeting specificity. In 4T1 tumor-bearing mice, M/A@MOF@CM + L achieved optimal tumor growth suppression. Flow cytometry analysis revealed that M/A@MOF@CM + L promoted DC maturation, increased CD8^+^ CTL infiltration, and reduced the proportion of Tregs. Furthermore, in bilateral breast tumor and lung metastasis models, M/A@MOF@CM + L suppressed tumor progression at both primary and metastatic sites while exhibiting minimal systemic toxicity. This study highlights the therapeutic potential of multifunctional nanoparticle-based platforms as next-generation cancer therapies.

Erastin (Er), a well-known ferroptosis inducer, has been demonstrated to exert an anti-Warburg effect through depletion of intracellular GSH and enhancement of lipid peroxidation, thereby sensitizing cancer cells to copper-induced cuproptosis while simultaneously triggering ferroptosis 159. Li et al. developed a novel core-shell nanoparticle, CuP/Er, consisting of a core loaded with copper ions and peroxides and a shell based on Er 160. Benefiting from EPR-mediated tumor accumulation and pH-responsive release of Cu^2+^ and Er in the acidic TME, CuP/Er achieved selective activation in tumor tissues. Upon internalized by tumor cells, CuP/Er promoted ferroptosis through ROS generation, lipid peroxidation, and GSH depletion. Concurrently, it induced cuproptosis *via* mitochondrial membrane disruption and glycolytic inhibition. *In vitro* experiments demonstrated that CuP/Er successfully induced DLAT aggregation and elevated lipid peroxidation in 4T1 cells, confirming the activation of both cuproptosis and ferroptosis pathways. Notably, CuP/Er elicited ICD, increasing the proportion of mature DCs from 12.3% (PBS control) to 19.8%. In the MC38 tumor model, CuP/Er exhibited a tumor growth inhibition rate of 86.5%, which was significantly higher than that achieved by CuP alone (73.9%), providing direct evidence that the induction of ferroptosis enhances the antitumor efficacy of cuproptosis. Compared to PBS control, CuP/Er administration elevated the percentage of intratumoral mature DCs from 9.2% to 24.0%, increased the proportion of M1 macrophages from 8.7% to 20.1%, and reduced M2 macrophage proportions from 34.0% to 7.7%. Furthermore, combination therapy with CuP/Er and αPD-L1 further enhanced intratumoral immune infiltration, increasing CD8^+^ T cell populations from 1.6% to 4.1% and CD4^+^ T cells from 0.7% to 5.6%, compared to the PBS control group. These findings collectively highlight a powerful strategy for eliciting robust antitumor immune responses and enhancing cancer immunotherapy efficacy through the co-activation of cuproptosis and ferroptosis pathways.

Dysregulation of copper metabolism in tumor cells limits copper accumulation and its associated cytotoxic effects. Additionally, elevated GSH levels and the hypoxic TME significantly impair the efficacy of cuproptosis-and ferroptosis-based therapies 161. To address these challenges, Gu et al. developed a nanoenzyme system, CussOMEp, composed of PEG-coated and omeprazole (an inhibitor of the copper transporter)-loaded copper-based nanoparticle carrier 162. This design endows the nanosystem with pH- and GSH-responsive degradability, thereby ensuring tumor-specific release of both copper ions and omeprazole. Release profiles demonstrated that under acidic and reductive conditions (pH 6.0 with 10 mM GSH), over 95% of copper and omeprazole were released. *In vitro* studies revealed that CussOMEp markedly elevated intracellular Cu^2+^ and •OH levels, depleted GSH, and induced lipid peroxidation. Western blot analysis showed that CussOMEp downregulated the expression of GPX4 and FDX1 and enhanced the oligomerization of DLAT, demonstrating the concurrent induction of ferroptosis and cuproptosis. In 4T1 tumor-bearing mice, CussOMEp monotherapy achieved a tumor growth inhibition rate of 71.6% on the day 14. Flow cytometry analysis showed that CussOMEp effectively reduced the proportion of M2 macrophages from 21.1% in the control group to 9.8%, suggesting a potent inhibitory effect on M2 macrophage polarization. Additionally, CussOMEp increased the percentage of mature DCs in the TDLNs to 21.8%, a substantial improvement compared to the control group. CussOMEp also enhanced the infiltration of CD4^+^ and CD8^+^ T cells. CussOMEp combined with αPD-1 resulted in more pronounced tumor suppression, with tumor growth almost ceasing. Notably, the combined treatment reduced the proportion of Tregs to 7.6% and further augmented T cell activation in distant tumors. All experiments were conducted with appropriate replicates and analyzed using one-way ANOVA. These findings highlight the potential of CussOMEp as a promising strategy for synergistic tumor immunotherapy by concurrently targeting ferroptosis and cuproptosis pathways.

### 4.11. Cuproptosis combined with gas therapy

Gas therapy has emerged as a promising antitumor strategy that primarily modulates the TME through the delivery of bioactive gas molecules, including H_2_, carbon monoxide, and hydrogen sulfide. Among these, H_2_ therapy has attracted significant attention due to its favorable properties, such as low molecular weight, high diffusibility, and excellent biological safety 163. The antitumor effects of H_2_ are primarily attributed to its ability to induce redox imbalance through mitochondrial dysfunction, selectively target tumor cells, modulate inflammatory factor secretion, and reshape the immunosuppressive TME 164. Despite these potential benefits, H_2_ therapy faces several challenges, including difficulties in concentration regulation, limited targeting delivery efficiency, and modest efficacy when administered as a monotherapy 165. Recent studies have explored the combination of H_2_ therapy with cuproptosis to achieve synergistic antitumor effects. On one hand, H_2_ reduces mitochondrial membrane potential and inhibits ATP synthesis, exacerbating copper ion-induced acylation and subsequent aggregation of TCA cycle proteins. On the other hand, cuproptosis-associated depletion of GSH impairs H_2_O_2_ clearance, thereby amplifying the oxidative stress mediated by H_2_. Preliminary studies have suggested that this combination strategy may activate the antitumor immune response, ultimately leading to improved tumor treatment outcomes.

Hydrogen selenide gas has demonstrated cytotoxic effects in cancer cells; however, its therapeutic efficacy as a standalone gas therapy remains limited. Zhao et al. developed an innovative nano-platform termed Cu_2_₋*_X_*Se@cMOF, which integrates copper selenide (Cu_2_₋*_X_*Se) with carbonized MOFs (cMOFs), enabling a tri-modal therapeutic approach that combines SDT, gas therapy, and cuproptosis induction 166. Mechanistically, Cu_2_₋*_X_*Se@cMOF achieves tumor selectivity through ultrasound-triggered and TME-responsive decomposition, thereby ensuring precise on-site release of copper and selenium ions for synergistic therapy. Flow cytometry demonstrated that 4T1 cells treated with Cu_2_₋*_X_*Se@cMOF and ultrasound exhibited a remarkably high apoptosis rate of 70.1%, underscoring the platform's potent therapeutic efficacy. Western blot analysis further revealed that Cu_2_₋*_X_*Se@cMOF downregulated the expression of Lip-DLAT, Lip-DLST, POLD1, and FDX1, providing evidence for the activation of cuproptosis pathways. Following intravenous administration in mice, Cu_2_₋*_X_*Se@cMOF accumulated specifically in tumor tissues, confirming their excellent tumor-targeting properties. *In vivo* studies using 4T1 tumor-bearing mice showed that Cu_2_₋*_X_*Se@cMOF + ultrasound + αPD-L1 significantly modulated the tumor immune microenvironment. Flow cytometry and ELISA indicated a marked increase in the levels of CD8^+^ T cells, NK cells, and M1 macrophage markers. Compared with the control group, the combination therapy effectively reduced the levels of Tregs and M2 macrophages. These findings suggest that Cu_2_₋*_X_*Se@cMOF effectively reshapes the tumor immune landscape, thereby enhancing antitumor immune responses.

### 4.12. Cuproptosis combined with electrodynamic therapy (EDT)

EDT represents a novel paradigm that harnesses external ultrasound or alternating electric fields to generate ROS through piezoelectric or triboelectric nanomaterials, thereby inducing oxidative stress in tumors 167. Compared to PDT, EDT exhibits superior tissue penetration and retains efficacy under hypoxic conditions. Key advantages of EDT include its non-invasive activation and precise spatiotemporal control. However, challenges persist regarding energy conversion efficiency, ROS specificity, and long-term material stability. Preclinical studies have demonstrated that EDT effectively triggers ICD, thereby activating DCs and CTLs 168. Despite these promising findings, tumor heterogeneity and inherent immunosuppressive mechanisms limit the therapeutic efficacy of EDT. To overcome these limitations, combination with other cell death modalities, such as cuproptosis, has been proposed as a potential strategy to enhance therapeutic outcomes. Mechanistically, EDT-derived ROS disrupt cellular redox balance and promote copper dysregulation, thus sensitizing tumor cells to cuproptosis 169. In turn, cuproptosis-induced mitochondrial dysfunction and protein aggregation increase oxidative susceptibility, amplifying EDT-mediated damage. This synergistic interaction not only enhances tumor cell killing but also strengthens ICD by eliciting robust release of DAMPs. Furthermore, EDT-mediated improvements in tumor oxygenation coupled with cuproptosis-driven proinflammatory signaling collectively promote CD8^+^ T-cell infiltration and reduce immunosuppressive myeloid cells 170. Overall, this feedback loop potentiates tumor eradication and advances nanomedicine-based immunotherapy.

Tumor tissues are often characterized by hypoxia, which significantly impair the therapeutic efficacy of dynamic therapies. To address these challenges, Wan et al. constructed a light-cured microneedle platform (N-PG/NSC) that co-delivers a nano-pomegranate carrier and Cu^2+^ ions, integrating EDT with cuproptosis induction for synergistic oral cancer treatment 170. The N-PG/NSC composite functions as an electrocatalyst for water splitting under an applied electric field, generating •OH, which are the dominant ROS in EDT. This mechanism circumvents the reliance on ambient O_2_ concentration. *In vitro* studies further revealed that N-PG/NSC + Cu^2+^ treatment substantially promoted DLAT oligomerization, verifying the occurrence of cuproptosis. In a subcutaneous SCC-7 tumor-bearing mouse model, the microneedle platform achieved precise intratumoral deposition *via* minimally invasive delivery with negligible leakage, thereby enhancing drug utilization and therapeutic efficacy. Notably, the combination of N-PG/NSC + Cu^2+^-loaded microneedles with external electric field stimulation resulted in the most pronounced tumor suppression among all experimental groups, as evidenced by the smallest tumor weights and the most significant growth inhibition. Moreover, flow cytometry analysis demonstrated that this integrated system effectively induced ICD in tumor cells and markedly enhanced DC recruitment, thereby amplifying antitumor immune responses. Collectively, these findings highlight the potential of combining EDT with cuproptosis induction *via* microneedle-mediated delivery to overcome barriers imposed by the TME and achieve synergistic enhancement of both direct cytotoxicity and immune activation against oral carcinoma.

### 4.13. Cuproptosis combined with glycolysis inhibition

Cuproptosis is inherently linked to mitochondrial metabolism, driven by copper binding to lipoylated TCA cycle proteins such as DLAT, which induces proteotoxic stress and subsequent cell death. Tumor cells exhibiting high reliance on aerobic glycolysis (the Warburg effect) display reduced TCA cycle flux, thereby diminishing mitochondrial copper accumulation and conferring resistance to cuproptosis 171. Inhibition of glycolysis or redirection of pyruvate into mitochondrial oxidation enhances oxidative phosphorylation activity, increasing the availability of lipoylated targets and amplifying copper-mediated cytotoxicity 172. This metabolic crosstalk holds significant immunological implications: reducing glycolytic flux not only sensitizes tumor cells to cuproptosis but also decreases lactate accumulation in the TME, thereby alleviating immunosuppression and promoting T cell infiltration. Consequently, the combinatorial therapeutic strategy employing cuproptosis inducers alongside glycolysis inhibitors or other metabolic modulators offers a synergistic approach to enhance tumor cell killing, promote ICD, and boost antitumor immunity.

Glycolytic dependence attenuates cuproptosis by diverting pyruvate from mitochondrial oxidation and promoting the accumulation of immunosuppressive lactate 173. To address this issue, Wu et al. developed a GSH-activated nanoassembly (Cu-GM) through coordination of copper ions with galloflavin (an LDH inhibitor) and myricetin (an immunomodulator) 174. The Cu-GM exhibited well-defined spherical nanostructures with a diameter of approximately 90 nm and a zeta potential of about -35 mV. These nanoassemblies demonstrated stability under physiological conditions and underwent disassembly in tumor-level GSH (3 mM), leading to the reduction of Cu^2+^ to Cu^+^ and the release of approximately 80% copper and 60% of each small-molecule component within 72 h. In PC-3 cells, Cu-GM effectively induced cuproptosis, as evidenced by DLAT aggregation and significant depletion of FDX1 and LIAS. Concurrently, galloflavin treatment resulted in reduced LDH activity and lactate levels. These cellular changes augmented ICD, as demonstrated *in vitro* by increased CRT exposure, HMGB1 release, and elevated extracellular ATP levels. In PC-3 tumor-bearing nude mice, flow cytometric analysis of splenocytes revealed that Cu-GM treatment significantly enhanced systemic immune activation, showing that the proportions of CD3^+^CD8^+^ T cells and CD3^+^CD4^+^ T cells were elevated by 3.4-fold and 3.3-fold, respectively, compared to the control group. Furthermore, the proportion of splenic Tregs were decreased by about 16.8%. In this study, all experiments were performed with biological replicates (n = 3-5 per group) and analyzed using Student's t-tests or one-way ANOVA with predefined significance thresholds (*P* < 0.05 or *P* < 0.01).

Building on the advances in nanomedicine and immunotherapy, Li et al. developed an innovative dual-remodeling cuproptosis-inducing nanoagent, termed DREAM, which combines copper-dependent toxicity with metabolic modulation to potentiate cancer treatment 175. This sophisticated nanoagent consists of a copper-doped mesoporous organosilicon core (co-loaded with DSF and the glycolysis inhibitor 3-bromopyruvic acid) and a fused cancer cell/M1 macrophage cell membrane shell, which not only enables homotypic tumor targeting but also potentiates immune activation. Transmission electron microscopy revealed that DREAM nanoparticles were quasi-spherical with an average diameter of 150 nm. *In vitro* studies employing confocal microscopy and flow cytometry demonstrated that DREAM exhibited significantly enhanced cellular uptake and deeper penetration into tumor spheroids compared to uncoated nanoparticles, confirming the functional role of the hybrid membrane in tumor targeting. DREAM effectively increased intracellular copper levels by 1.81-fold relative to a conventional cuproptosis-inducing agent and by 1.90-fold compared to a dual-remodeling nanoagent lacking the hybrid membranes (DREA), thereby triggering hallmark features of cuproptosis. Concurrently, DREAM inhibited glycolysis, as evidenced by reductions in lactate, glucose-6-phosphate, and pyruvate levels, achieving robust metabolic remodeling. In a bilateral SCC7 murine squamous cell carcinoma model, DREAM reduced primary tumor volume from 938.51 ± 158.45 mm^3^ (control group) to 199.24 ± 117.52 mm^3^. Flow cytometry analysis revealed robust immune activation, with DC maturation rates significantly increasing from 18.44 ± 3.08% to 50.96 ± 5.09% in primary tumors and from 16.72 ± 2.79% to 43.14 ± 5.11% in distant tumors (n = 5, one-way ANOVA, *P* < 0.001). This immune response was accompanied by enhanced CD8^+^ T-cell activity, a critical component of antitumor immunity. Furthermore, DREAM effectively remodeled the TME by increasing the proportion of M1 TAMs and reducing M2 TAMs and Tregs in both primary and distant lesions. Collectively, DREAM synergizes metabolic restraint and copper-dependent lethality to reprogram the TME from immune-cold to immune-hot, providing a potent strategy for cuproptosis-based immunotherapy.

Recent advances in cancer nanomedicine have focused on developing multifunctional nanoagents that can simultaneously modulate cellular metabolism and activate antitumor immunity. Zhang et al. developed a self-reinforcing metabolic nanojammer, Hb/Mito-DCA@Cu/ZIF-8@HA (HMCZH), constructed from a copper-doped zeolite imidazole framework (ZIF-8) core co-loaded with oxygenated hemoglobin (Hb) and mitochondria-targeted dichloroacetate (Mito-DCA), and subsequently encapsulated in a HA layer for enhanced CD44-mediated tumor uptake 176. This nanoformulation demonstrated enhanced cellular uptake in 4T1 cancer cells compared to HUVECs, as evidenced by confocal microscopy and flow cytometry analyses. At the metabolic level, HMCZH induced a significant shift in cellular bioenergetics, promoting oxidative phosphorylation while suppressing glycolysis. The ratio of ATP produced by oxidative phosphorylation to that produced by glycolysis has increased by 155%, accompanied by a marked reduction in extracellular lactate levels, which decreased by 19.69% at 24 h and further dropped to 36.55% at 48 h. Furthermore, JC-1 assays revealed a dissipation of mitochondrial membrane potential, indicative of mitochondrial dysfunction. HMCZH triggered cuproptosis by depleting intracellular GSH by up to approximately 88% and suppressing Cu-ATPase activity by about 83% at 12 h. *In vivo* studies using the 4T1 tumor model demonstrated robust immune reprogramming. Flow cytometry analysis of tumor tissues revealed a remarkable enhancement in antitumor immunity under HMCZH treatment compared to PBS control: mature DCs increased from 6.17% to 28.9%, intratumoral CD8^+^ T cells rose from 2.88% to 8.78%, and CD4^+^ T cells expanded from 2.56% to 13.9%. Additionally, TAMs underwent significant repolarization, with the proportion of M1 macrophages reaching 18.6% and the proportion of M2 macrophages declining to 3.9%, representing the most favorable immune profile among all tested nanoformulations. These findings highlight the potential of HMCZH as a novel nanoagent for cancer immunotherapy through its unique ability to integrate metabolic intervention with immune activation.

The initiation of cuproptosis depends on the accumulation of copper ions in mitochondria and active mitochondrial respiration. Since most tumors rely on anaerobic glycolysis rather than mitochondrial respiration to meet their large energy demands, inhibiting glycolysis helps boost cuproptosis. To targe mitochondria and anaerobic glycolysis synergistically, Zhang et al. self-assembled the lactate dehydrogenase inhibitor galloflavin with the copper ion carrier ES through copper ion-driven synergistic coordination to prepare GF/CuES hybrid nanoparticles 177. *In vitro* experiments showed that GF/CuES effectively inhibited the proliferation of MCF-7 cells, while the introduction of cuproptosis inhibitor significantly improved the survival rate of the cells, indicating that cuproptosis is the main mechanism of cytotoxicity of GF/CuES against tumor cells. Moreover, GF/CuES significantly reduced lactate dehydrogenase activity and lactate levels, indicating the effective blockade of glycolysis in tumor cells *via* LDH inhibition. *In vivo* optical imaging showed that GF/CuES nanoparticles accumulated at the tumor site for up to 24 h, confirming their effective tumor-targeting and long-term retention capabilities. In the subcutaneous MCF-7 tumor model, the tumor inhibition rate of GF/CuES was 93.9%, markedly higher than that of CuES (31.3%). Flow cytometry analysis showed that the infiltration of CD4^+^ T cells and CD8^+^ T cells in the GF/CuES group was significantly higher than that in other groups. The levels of TNF-α, IFN-γ, and interleukin-6 in the tumor tissues of the GF/CuES group were 3.6-, 3.0-, and 5.02-fold those of the control group, respectively. These findings suggest that GF/CuES is a promising approach for promoting immune-metabolic reprogramming.

Combining the induction of cuproptosis with glycolysis inhibition represents a highly promising therapeutic strategy to enhance copper-dependent cytotoxicity and therapeutically modulate the tumor 178. By simultaneously targeting key metabolic vulnerabilities and promoting activation and infiltration of various immune cell populations, this approach establishes cuproptosis-based therapy as a potential strategy for advancing next-generation cancer immunotherapy.

## 5. Conclusions and perspectives

Programmed cell death is a genetically regulated and highly ordered process essential for maintaining cellular homeostasis and preventing diseases. In recent years, various forms of programmed cell death beyond apoptosis, such as necroptosis, autophagy, pyroptosis, ferroptosis, and cuproptosis, have been progressively identified and characterized. Cuproptosis, characterized by copper-dependent aggregation of lipoylated proteins and mitochondrial dysfunction, displays unique features that distinguish it from other forms of cell death. Importantly, tumor cells undergoing cuproptosis release DAMPs and pro-inflammatory cytokines, which promote DC maturation, enhance CD8^+^ T cell and NK cell infiltration, and reduces the proportion of immunosuppressive cells 35. These findings underscore cuproptosis as a promising mechanism for reprogramming the immune milieu and enhancing the efficacy of immunotherapy. Accumulating evidence demonstrates that integrating cuproptosis with chemotherapy, RT, ICIs, PDT, PTT, CDT, and SDT, and other therapeutic modalities can markedly enhance antitumor outcomes. For example, Sun et al. demonstrated that simultaneous induction of cuproptosis and ferroptosis yields synergistic tumoricidal effects and triggers ICD, thereby augmenting T cell-mediated immune responses 179. However, the potential risk of cuproptosis induction in normal tissues underscores the necessity for precision strategies that confine copper accumulation and associated cell death to tumor sites.

Nanotechnology offers promising avenues for expanding the applications of cuproptosis in cancer immunotherapy. To date, a variety of nanomaterials have been engineered to induce cuproptosis and stimulate antitumor immune responses (Table 1). Nanomaterials have demonstrated numerous advantages in enhancing cuproptosis-based immunotherapy. Firstly, they enable the targeted accumulation of copper ions at tumor sites through both passive targeting *via* the EPR effect and innovative active targeting strategies, allowing precise and controlled delivery to tumor tissues, cells, or specific organelles while minimizing off-target toxicity. For instance, Noh et al. developed bovine serum albumin-based nanoparticle carriers, which are preferentially taken up by tumor cells and exhibit enhanced accumulation in solid tumors compared to normal tissues 87. Additionally, hydroxyethyl starch further enhances tumor targeting through interactions with tumor cell surface molecules. Secondly, the unique characteristics of the TME, such as acidity, hypoxia, redox imbalance, and elevated enzyme activity, can be leveraged by stimuli-responsive nanomaterials to achieve selective copper ion release within tumor tissues or cells. This targeted and controlled delivery enhances therapeutic efficacy while ensuring safety. For example, Li et al. developed a dual-responsive nanocomposite (CytoNano) that responds to both acidic pH and ultrasound, enabling precise tumor targeting, improving therapeutic outcomes, and reducing collateral damage 180. In acidic environments, CytoNano strategically releases copper ions that preferentially target cancer cells and mitochondria, promoting the synergistic effect of SDT and cuproptosis. Thirdly, given the limitations of monotherapies, nanomaterials provide an excellent platform for integrating cuproptosis into multimodal therapeutic strategies. Hu et al. constructed a copper-doped BiSex nanozyme that induces ICD through photothermal effects, peroxidase-like activity, and cuproptosis, significantly enhancing antitumor immune responses and demonstrating substantial therapeutic potential 181. Fourthly, nanocarriers can improve the bioavailability and systemic stability of drugs with poor solubility, limited stability, or short biological half-lives, thereby broadening their clinical applicability. Finally, some nanomaterials exhibit superior photoacoustic or magnetic resonance imaging capabilities, enabling simultaneous tumor visualization and real-time monitoring of therapeutic responses 182. In the future, the development of multifunctional nanomaterials that integrate tumor and organelle targeting capabilities, TME-responsive copper release, multimodal designs to reinforce ICD, and imaging-guided monitoring systems will be pivotal in advancing cuproptosis-based cancer therapies 183. These innovative strategies are specifically engineered to address the fundamental challenges currently hindering the precise application of cuproptosis, including the achievement of spatial selectivity at both tissue and cellular levels, the maximization of on-target therapeutic potency while significantly reducing systemic copper exposure and associated off-target effects, as well as the implementation of real-time assessment systems for monitoring therapeutic responses. By overcoming these critical barriers, such nanomaterials will not only enhance the safety and efficacy of cuproptosis-based interventions but also pave the way for their successful clinical translation.

When contextualized within the broader spectrum of biomaterial platforms, nanomaterials exhibit distinct advantages over alternative materials like gels and organic molecules. Specifically, nanomaterials demonstrate superior performance in systemic delivery, organelle-specific targeting, and multimodal integration, making them particularly suitable for treating disseminated or metastatic diseases. These nanocarriers leverage their unique physicochemical properties to achieve precise spatiotemporal control over therapeutic interventions, which is crucial for minimizing off-target effects while maximizing therapeutic efficacy. In contrast, gels demonstrate remarkable utility in localized and sustained drug release, particularly in post-surgical tumor beds, where their ability to provide a controlled release of therapeutic agents can enhance treatment outcomes. However, the clinical application of gels is constrained by their limited tissue penetration and inability to address systemic or metastatic conditions effectively 184. Organic molecules offer advantages in terms of synthesis simplicity and standardization, but they often lack the spatial precision required for targeted therapies 185. This limitation increases the likelihood of off-target toxicity, which poses significant challenges in clinical translation. The integration of these biomaterial platforms in a context-specific manner represents a promising strategy to expand the therapeutic scope of cuproptosis-based interventions. By harnessing their unique physicochemical properties and tailoring their application to specific clinical scenarios, it is possible to develop more effective and precise treatment modalities for various diseases.

Although nanomaterial-mediated cuproptosis treatment strategy represents a promising strategy for cancer immunotherapy, several critical challenges remain to be addressed. Firstly, the molecular triggers and signaling pathways of cuproptosis remain incompletely understood, particularly regarding its interplay with mitochondrial metabolism, immune activation, and other regulated cell death pathways 186. The lack of reliable biomarkers further constrains patient stratification and therapeutic monitoring. Systematic approaches, such as CRISPR-Cas9 screening and metabolomics, are needed to elucidate underlying mechanisms and identify predictive indicators 187. Secondly, tumor metabolic adaptations, such as elevated GSH levels and differential expression of copper transporters (CTR1, ATP7A/B), limit intracellular copper accumulation, thereby attenuating cuproptosis induction 188. Strategies including GSH-responsive carriers, co-delivery of GSH-depleting agents, and modulation of copper transport pathways have shown promise in overcoming these barriers. For instance, Liu et al. engineered a nanosystem (CaCO_3_/Mn/Cu@lip-Apt) that released Mn^2+^ ions to significantly deplete intracellular GSH levels, thereby promoting Cu^2+^ accumulation and enhancing the efficacy of cuproptosis 189. Thirdly, the therapeutic window of copper is narrow, necessitating safe encapsulation and controlled release to minimize systemic toxicity while achieving effective tumor-specific cytotoxicity 190. Overcoming *in vivo* barriers, such as rapid clearance, vascular endothelial barriers, dense tumor stroma, and elevated interstitial fluid pressure, requires multifunctional nanocarriers that integrate passive and active targeting, charge optimization, extracellular matrix-remodeling modules, and size-transformable designs 191. Fourthly, the immunosuppressive TME, characterized by hypoxia, rich in immunosuppressive cells, and high levels of inhibitory cytokines, constrains the immune-potentiating effects of cuproptosis. Combining cuproptosis induction with oxygen-generating materials or ICIs constitutes a rational strategy to enhance immune responses and overcome these suppressive barriers. In addition, although combining cuproptosis with established therapies, such as chemotherapy, RT, PDT, and ICIs, shows promise, the molecular basis of both synergy and toxicity remains poorly defined. Optimizing dosing sequences, minimizing overlapping toxicities, and developing predictive models of immune response dynamics represent essential steps toward the rational design of combination regimens 192.

Finally, despite significant advancements in developing nanomaterials capable of inducing cuproptosis, their clinical translation remains hindered by numerous unresolved challenges. Among these, long-term safety, biocompatibility, targeting precision, *in vivo* stability, absence of biomarkers, and scalability issues in large-scale production are particularly prominent concerns 186. Regarding biosafety, preclinical studies predominantly rely on short-term assessments, such as alterations in body weight, serum biomarker levels, and organ histopathology in animal models. However, these methods fail to provide insights into the long-term accumulation of nanomaterials or their potential chronic toxicological effects. Ensuring *in vivo* biocompatibility is crucial for clinical applications, as excessive accumulation of nanomaterials may lead to oxidative stress, inflammatory responses, or toxicity to vital organs like the liver and kidneys 193. Furthermore, achieving efficient delivery to specific target tissues or cell populations is essential for maximizing therapeutic efficacy, yet this remains a significant challenge in the field. Surface modification, size optimization, and functional design of nanomaterials are critical strategies to enhance targeting accuracy and delivery efficiency 194. Another critical factor influencing therapeutic performance is the *in vivo* stability of nanomaterials. In biological environments, these materials often undergo degradation or interactions with biomolecules, which can compromise their stability and diminish therapeutic effectiveness 195. The absence of cuproptosis-related biomarkers not only complicates patient selection and therapeutic monitoring but also hinders the rational design and mechanistic validation of nanomedicines, thereby increasing the uncertainty and risk of clinical trial failures 196. Additionally, the development of complex multifunctional nanomedicines faces substantial challenges in large-scale production, quality control, cost-effectiveness, and regulatory approval processes 197. Recent advancements in artificial intelligence (AI) technologies have opened new avenues for optimizing nanomaterial design, preclinical research, targeted delivery strategies, clinical trial management, and production workflows. These AI-driven innovations hold the potential to accelerate the research and development cycle, enhance therapeutic efficacy, mitigate toxicity risks, and facilitate the rapid translation of nanomedicines into clinical practice, offering promising prospects for cancer treatment 198.

In summary, the mechanisms underlying cuproptosis and its associated immune activation are being progressively elucidated. Notably, nanomaterials have demonstrated remarkable potential in synergizing cuproptosis with other antitumor therapies to amplify immunogenic responses. Given the rapid advancements of nanotechnology in cancer treatment, cuproptosis emerges as a promising frontier with significant potential for clinical translation and may serve as a cornerstone in future tumor immunotherapy. Therefore, research in this field not only opens innovative avenues for cancer therapy but also paves the way for the development of more precise and effective immunotherapeutic strategies.

## Figures and Tables

**Table 1:** Summary of representative nanosystems for cuproptosis-based combined antitumor immunotherapy.

Synergistic Therapy	Nanosystem	Cuproptosis inducers	Cancer type	References
Chemotherapy	GPCuD NPs PCD@Cu	Cu^2+^ Cu^2+^	4T1 4T1	98 97
RT	CHP RCL@Pd@CuZ ^Cu/AP^H-M	Cu^2+^ Cu-MOF Cu^2+^	4T1 MC38 CT26	103 102 101
ICIs	NP@ESCu CMGCL Pt/Cu DAzyme TSF@ES-Cu NPs OMP CAT-ecSNA-Cu	Cu^2+^, ES Cu^2+^ Cu^2+^ Cu^2+^, ES Cu^2+^ Cu^2+^	MB49 LLC CT26 Pan02 B16F10 CT26	55 112 106 114 22 65
PDT	CCNAs NPs	Cu^2+^ Cu^2+^	PC-3 LLC	118 116
PTT	PCD@CM CEL NP AHPR PEG@Cu_2_O-ES	Cu^2+^ Cu^2+^, Cu_2_₋*_X_*S, ES CuS Cu_2_O	4T1 CT26 4T1 4T1	124 126 125 66
CDT	mCGYL-LOx HA-CD@MOF ECNM	Cu^2+^ Cu^2+^ Cu^2+^, ES	Renca 4T1 4T1	132 133 135
SDT	ZCA NSs SPN_LCu_ CMC CRUPPA19	Cu^2+^ Cu^2+^ Cu_2_O, Cu-MOF Cu^2+^	CT26, 4T1 Panc02 4T1 A20	139 141 144 143
Pyroptosis	F^127^MOF-199 NPs OCT@ES CKPP Cu-Pic/HA NPs CQG NPs	Cu^2+^, Cu_2_₋*_X_*S Cu^2+^, ES Cu^2+^ Cu^2+^ Cu^2+^	CT26 Hepa1-6, CT26 CT26 4T1 4T1	149 150 148 153 91
Ferroptosis	CuP/Er CussOMEp M/A@MOF@CM	Cu^2+^ Cu^2+^ Cu-MOF	MC38, 4T1 4T1 4T1	160 162 29
Gas therapy	Cu_2_₋*_X_*Se@cMOF	Cu_2_₋*_X_*Se	4T1	166
EDT	N-PG/NSC	Cu^2+^	SCC-7	170
Glycolysis inhibition	Cu-GM DREAM HMCZH GF/CuES	Cu^2+^ Cu^2+^ Cu^2+^ Cu^2+^, ES	PC-3 SCC-7 4T1 MCF-7	174 175 176 177
